# Zika virus infection of mature neurons from immunocompetent mice generates a disease-associated microglia and a tauopathy-like phenotype in link with a delayed interferon beta response

**DOI:** 10.1186/s12974-022-02668-8

**Published:** 2022-12-20

**Authors:** Caroline Manet, Zeyni Mansuroglu, Laurine Conquet, Violaine Bortolin, Thomas Comptdaer, Helena Segrt, Marie Bourdon, Reyene Menidjel, Nicolas Stadler, Guanfang Tian, Floriane Herit, Florence Niedergang, Sylvie Souès, Luc Buée, Marie-Christine Galas, Xavier Montagutelli, Eliette Bonnefoy

**Affiliations:** 1 Institut Pasteur, Mouse Genetics Laboratory, Université de Paris, 75015 Paris, France; 2 Université Paris Cité, Institut Cochin, Inserm, CNRS, 75014 Paris, France; 3 University Lille, Inserm, CHU Lille, Inserm, LilNCog - Lille Neuroscience & Cognition, 59000 Lille, France; 4 Université Paris Cité, Inserm UMR1124, 75006 Paris, France

**Keywords:** Zika virus, Interferon beta, Tau protein, Collaborative cross, Disease associated microglia, Neuron-microglia crosstalk, Type I interferon signaling, Microglia activation, Neuronal viral infection, Flavivirus

## Abstract

**Background:**

Zika virus (ZIKV) infection at postnatal or adult age can lead to neurological disorders associated with cognitive defects. Yet, how mature neurons respond to ZIKV remains substantially unexplored.

**Methods:**

The impact of ZIKV infection on mature neurons and microglia was analyzed at the molecular and cellular levels, in vitro using immunocompetent primary cultured neurons and microglia, and in vivo in the brain of adult immunocompetent mice following intracranial ZIKV inoculation. We have used C57BL/6 and the genetically diverse Collaborative Cross mouse strains, displaying a broad range of susceptibility to ZIKV infection, to question the correlation between the effects induced by ZIKV infection on neurons and microglia and the in vivo susceptibility to ZIKV.

**Results:**

As a result of a delayed induction of interferon beta (IFNB) expression and response, infected neurons displayed an inability to stop ZIKV replication, a trait that was further increased in neurons from susceptible mice. Alongside with an enhanced expression of ZIKV RNA, we observed in vivo, in the brain of susceptible mice, an increased level of active Iba1-expressing microglial cells occasionally engulfing neurons and displaying a gene expression profile close to the molecular signature of disease-associated microglia (DAM). In vivo as well as in vitro, only neurons and not microglial cells were identified as infected, raising the question of the mechanisms underlying microglia activation following brain ZIKV infection. Treatment of primary cultured microglia with conditioned media from ZIKV-infected neurons demonstrated that type-I interferons (IFNs-I) secreted by neurons late after infection activate non-infected microglial cells. In addition, ZIKV infection induced pathological phosphorylation of Tau (pTau) protein, a hallmark of neurodegenerative tauopathies, in vitro and in vivo with clusters of neurons displaying pTau surrounded by active microglial cells.

**Conclusions:**

We show that ZIKV-infected mature neurons display an inability to stop viral replication in link with a delayed IFNB expression and response, while signaling microglia for activation through IFNs-I secreted at late times post-infection. In the brain of ZIKV-infected susceptible mice, uninfected microglial cells adopt an active morphology and a DAM expression profile, surrounding and sometimes engulfing neurons while ZIKV-infected neurons accumulate pTau, overall reflecting a tauopathy-like phenotype.

**Supplementary Information:**

The online version contains supplementary material available at 10.1186/s12974-022-02668-8.

## Background

Zika virus (ZIKV) is a human, neurotropic, mosquito-borne flavivirus [1] first identified in 1947 in Uganda. After remaining mostly unnoticed for over 50 years, causing seroconversions and only sporadic mild febrile symptoms [2], it was responsible for a first mild outbreak on Yap island in 2007 and for a much larger epidemic in French Polynesia in 2013. It emerged in South America in 2015 where it spread to over 20 countries and is now endemic in over 80 countries worldwide (https://www.who.int/health-topics/zika-virus-disease#tab=tab_1). During these last two outbreaks, a significant increase of neuropathologies was reported, including Guillain-Barré Syndrome (an autoimmune inflammatory disease of the peripheral nervous system) in adults [3] and microcephaly in newborns [4]. These neuropathies were proven to be strongly associated with peaks of infection [3, 5, 6] and experimental evidence, particularly using animal models, demonstrated the causative role of ZIKV [7].

Until recently, microcephaly in newborns was the most abundantly described ZIKV-induced neuropathology and thus fetal neural progenitor cells rather than mature neurons have been considered as the main target of infection. However, there is now evidence in humans [8–10], macaques [11, 12] and mice [13, 14] that delayed neuropathies of the central nervous system (CNS), associated with cognitive disorders, can develop following neonatal and adult ZIKV infections.

At the cellular level, ZIKV infection of hippocampal slice cultures from newborn mice has provided evidence that ZIKV infects and induces damage in neurons independently of their differentiation (maturation) state [15]. ZIKV also replicates in neurons in adult human cortical tissue [14] and impacts human brain organization and structure [16]. Similar observations were reported after intracranial (IC) inoculation to adult mice where ZIKV displayed a tropism for mature neurons [14]. At the molecular level, the innate antiviral type I interferons (IFNs-I) response, mediated by interferon beta (IFNB) and alpha (IFNA), inhibits ZIKV replication and protects against infection in wild type mice following peripheral inoculation [17]. However, even though IFNs-I modulate ZIKV replication, they do not protect against ZIKV infection in the brain of adult wild type mice after IC inoculation [18].

Recognition of viral nucleic acids by Pattern-Recognition Receptors (PRRs) [19] leads to the rapid transient up-regulation of the expression of the *Ifnb1* gene coding for IFNB. After binding to its receptor IFNAR present on the surface of most cell types (including neurons), IFNB induces the transient expression of a large set of interferon stimulated genes (ISGs). Among them, the gene coding for transcription factor IRF7, that is constitutively expressed only in plasmacytoid dendritic cells (pDCs), whose expression is induced in many cell types following activation of IFNAR signaling. Upon phosphorylation, IRF7 triggers the production of IFNAs that, once secreted, bind to IFNAR amplifying the response initially triggered by IFNB [19, 20]. While the IFNB response plays a pivotal role stopping viral replication and propagation [20], its deregulation can promote brain inflammation and cellular lesions in association with the development of neurological disorders [21–23]. How mature neurons respond to ZIKV infection, in particular with respect to the innate immune response, remains to be established.

To shed light on the mechanisms of post-natal ZIKV-induced CNS damage, we have analyzed the impact of ZIKV infection on mature neurons from immunocompetent mice that, contrary to immuno-compromised mice often used in the context of ZIKV infection [24], have an intact IFN-I pathway. The impact of ZIKV infection was analyzed at the molecular and cellular levels, in vitro using primary cultured neurons (PCN) after they have reached maturation, and in vivo in the brain of adult mice following IC inoculation. We have taken advantage of our recent characterization of genetically diverse Collaborative Cross (CC) mouse strains displaying a broad range of susceptibility to ZIKV infection [25] to question the potential correlation between the effects induced by ZIKV infection in cultured mature neurons and the in vivo susceptibility to ZIKV. We focused on the capacity of neurons to set up an efficient IFNB response in link with microglia activation and the pathological phosphorylation of Tau protein (pTau) that alongside with the presence of diseased associated microglia (DAM) [26, 27], is a hallmark of tauopathies, a wide range of neurodegenerative diseases including Alzhemer’s disease (AD) associated with cognitive disorders [28–30].

## Materials and methods

### Virus

The FG15 Asian ZIKV strain, isolated from a patient during a ZIKV outbreak in French Guiana in December 2015, was obtained from the Virology Laboratory of the Institut Pasteur of French Guiana. Viral stocks were prepared from the supernatant of infected C6/36 cells, clarified by centrifugation and titrated on Vero cells (ATCC CRL-1586) by a focus-forming assay (FFA). Stocks were kept at − 80 °C. All experiments with ZIKV were carried out in biosafety level 3 facilities.

### Mice

C57BL/6J (B6) mice (purchased from Charles River Laboratories France) and Collaborative Cross CC001 and CC071 mice (purchased from the Systems Genetics Core Facility, University of North Carolina and bred at the Institut Pasteur) were maintained under specific-pathogen-free conditions with a 14-h light and 10-h dark cycle and ad libitum food and water in the Institut Pasteur animal facility. In all experiments, mice were killed by cervical dislocation. All experimental protocols were approved by the Institut Pasteur Ethics Committee (dap190107) and authorized by the French Ministry of Research (#19469), in compliance with French and European regulations.

### Antibodies

Primary antibodies used for immunofluorescence and/or Western blot (WB) were: anti-GSK-3α/β (sc-7291), anti-pGSK-3α/β (sc-81496) and anti-βActin (sc-47778) from SantaCruz; anti-MAP2 (188002) from Synaptic Systems; anti-beta tubulin III, TUJ1 (MAB 1637) from Millipore; anti-pTau antibodies: AT8 (MN1020) and AT100 (MN1060) from ThermoFisher and AD2 from the laboratory Alzheimer & Tauopathies (LilNCog, Lille, France) [31]; anti-NeuN (GTX16208) and anti-ZIKV protein NS2B (GTX133308) from GeneTex; anti-Iba1 (5076) from Abcam and (019-19741) from Wako. Secondary antibodies used for immunofluorescence were Alexa 488 fluor-conjugated chicken anti-mouse (A21200) and Alexa 555 fluor-conjugated donkey anti-rabbit (A31572) from Invitrogen. Secondary antibodies used for WB were ECL Mouse IgG, HRP-linked whole Ab (NA931) and ECL Rabbit IgG, HRP-linked whole Ab (NA934) from GE Healthcare Life Sciences. Anti-IFNARα/β antibody (I-401, Leinco Technologies) was used for neutralization of type I IFN(α/β) response.

### Mouse ZIKV intracerebral infection

All infection experiments were performed in a biosafety level 3 animal facility as described previously [25]. After being anesthetized by intraperitoneal (IP) injection of Ketamine 70 mg/kg and Xylazine 5 mg/kg, groups of 5- to 6-week-old mice were inoculated by IC injection. Mice received either 10^5^ foci forming units (FFUs) of ZIKV FG15 in phosphate-buffered saline (PBS) or PBS alone in a volume of 10 µl. Survival and clinical signs were monitored daily for 6 days, and the mice were euthanized for brain collection.

### Primary neuronal culture and infection

Primary cortical neurons were prepared from individual fetuses from two or more genetically identical females at day 15.5 and 16.5 of gestation. Cortexes from 6 to 8 fetuses/female were carefully dissected out, incubated for 15 min with Trypsin (T6763, Sigma) and DNAse I (11284932001, Sigma) at 1 and 0.5 mg/ml final concentration respectively and mechanically dissociated by triturating with a polished Pasteur pipette. Each embryo was dissected individually and cells from the different fetuses were mixed before plating. Neurons were maintained in Neurobasal medium supplemented with B27 (17504-044, Gibco), l-glutamine (25030-024, Gibco) and antibiotic/antimycotic (15240-096, Gibco). Tissue culture plate wells and coverslips for immunofluorescence microscopy were coated with poly-d-lysine 0.1 mg/ml (P7280, Sigma) and laminin 20 µg/ml (L2020, Sigma) final concentrations respectively. For RNA extraction, cells were plated in 6-well plate with 600,000 cells/well. For immunofluorescence and WB, cells were plated in 12-well plates with 150,000 and 280,000 cells/well respectively. Cells were maintained at 37 °C and 5% C02 with one third of the culture medium changed every 5–6 days. Primary neuron cultures were infected at a multiplicity of infection (MOI) = 5 at 11 days of in vitro (DIV) culture. For infection, the whole medium was removed and replaced with 1 ml of virus solution. After 1 h infection, the inoculum was removed and new culture medium (1/2 fresh and 1/2 old) was added. Cells isolated from the same dissection were cultured in parallel in 6 well plates for RNA collection and in 12 well plates for immunofluorescence. All cells were infected at the same time with the same virus-containing medium. At the indicated times, cells in 6 well plates were collected using the RNeasy Plus kit (Qiagen) and cells in the 12 well plates were fixed with 3.7% formaldehyde in PBS for 15 min. Supernatants were collected for titration at the indicated times post-infection (p.i.). When indicated, anti-IFNAR antibody or recombinant IFNB (8234-MB, R&D Systems) were added in the culture medium overnight before infection and 48 h p.i. at a final concentration of 6.5 µg/ml and 50 U/well respectively.

### Primary culture of microglia and infection

Primary microglia were prepared from pools of cortices from 0- or 1-day-old newborn B6 mice (*n* = 12 newborns per culture). After decapitation, hemispheres were dissected and stripped of meninges, olfactory bulbs and cerebellum. Cortices were incubated at 37 °C with 0.25% trypsin (25200-056, Gibco) for 15 min and with 0.6 mg/ml final concentration of DNAse I (11284932001, Sigma) for 5 min and then mechanically dissociated by triturating with a polished Pasteur pipette. After dissociation, glial cells were plated into poly-l-ornithine- (P3655, Sigma) coated Petri dishes containing glial culture medium: DMEM (31885, Gibco) supplemented with 10% heat-inactivated fetal bovine serum (FBS, Gibco), 0.1% penicillin–streptomycin (15140-148, Gibco) and 1% l-Glutamine (25030-024, Gibco). Cells were maintained in incubators at 37 °C 5% CO_2_ and medium was replaced at 1 and 3 DIV. Microglia were detached from the astrocytic layer after 13 DIV by gentle shaking and plated in 12-well plates with 600,000 cells/well for RNA extraction and in 24-well plates containing coverslips with 100,000 cells/well for immunofluorescence. Cells were maintained in glial culture medium at 37 °C and 5% C0_2_ and infected 1 day after plating. For infection, the whole medium was removed and replaced with 0.5 ml of either glial culture medium, conditioned media collected from non- or ZIKV-infected PCNs or medium containing the indicated amounts of ZIKV viral particles. When indicated, anti-IFNAR antibodies were added overnight before infection at 2.6 µg/ml final concentration.

### MEF isolation and infection

Mouse embryonic fibroblasts (MEFs) were isolated from individual fetuses from one or more genetically identical females at day 13.5 to 15.5 of gestation. Embryo head and liver were removed, and the embryo body was minced into small pieces in a Petri dish using scalpels. The mixture was trypsinized in a 7 mL volume for 15–20 min at 37 °C. The supernatant was added to 7 mL of DMEM (Gibco) supplemented with 10% FBS (Eurobio) and 1% penicillin–streptomycin (Gibco) and centrifuged for 5 min at 300 rcf. The pellet was suspended and cultured in DMEM supplemented with 10% FBS and 1% penicillin–streptomycin at 37 °C. MEFs were used until passage 2. For RNA extraction and WB, MEFs were plated in 6-well plates with 200,000 cells/well and for immunofluorescence, in 24-well plates containing coverslips with 100,000 cells/well. Twenty-four hours after plating, cells were infected with ZIKV FG15 at an MOI of 5. For infection, the whole medium was removed and replaced with 1 ml of virus solution. After 2 h incubation at 37 °C, the inoculum was replaced with fresh medium. Supernatants were collected for titration at the indicated times p.i.

### Focus-forming assay (FFA)

Viral titration was performed as described previously [25] using Vero cells seeded at 30,000 cells per well in 100 µl DMEM (Gibco) supplemented with 10% FBS (Eurobio). After overnight incubation at 37 °C, the medium was replaced with 40 µl of serial tenfold dilutions of the samples, and 115 µl of methylcellulose overlay was added 2 h later. After 40 h of incubation, the cells were fixed with 4% paraformaldehyde for 20 min and permeabilized with a solution of 0.3% Triton X-100 and 5% FBS in PBS for 20 min. The cells were washed and incubated with a mouse MAb directed against ZIKV envelop protein (MAb 4G2, ATCC) for 1 h at 37 °C (1/250 in blocking buffer). The cells were further washed, incubated with a secondary antibody (Alexa Fluor 488-conjugated anti-mouse IgG; Invitrogen) for 45 min at 37 °C. Infected cell foci were counted using ImmunoSpot CTL analyzer, and viral titers were calculated from the average number of foci.

### RT-qPCR

Total RNA from primary cultured neurons, microglia and MEFs was extracted using the RNeasy Plus kit (Qiagen). Brain tissue samples were homogenized at 4 °C in 1 ml of Trizol reagent (Life technologies), using ceramic beads and an automated homogeniser (PreCellys). RNA was reversely transcribed using High Capacity cDNA Reverse Transcription Kit (Applied Biosystems) according to the manufacturers' recommendations using Random Primers. qPCR was performed using SENSIFAST SYBR NO-ROX MIX (Bio-TechnoFix) reagents at: 95 °C 2 min, then 40 cycles at 95 °C 5 s, 60 °C 10 s, 72 °C 15 s, followed by a dissociation step. Relative quantification of mRNA expression was calculated by the *∆C*_*T*_ method using two reference genes: *Rplp0* and *Ppib* for PCNs, *Hrpt1* and *Utp6c* for brain extracts and *Rplp0* and *Utp6c* for primary cultured microglia. For each condition, the quantifications calculated with one of the two reference genes are shown. Sequences (5′–3′) of primers used for qPCR analysis are listed in Additional file 6: Table S1.

### Immunofluorescence, image analysis and quantification

#### Primary cultured neurons, microglia and MEFs

**Fig. 1:**
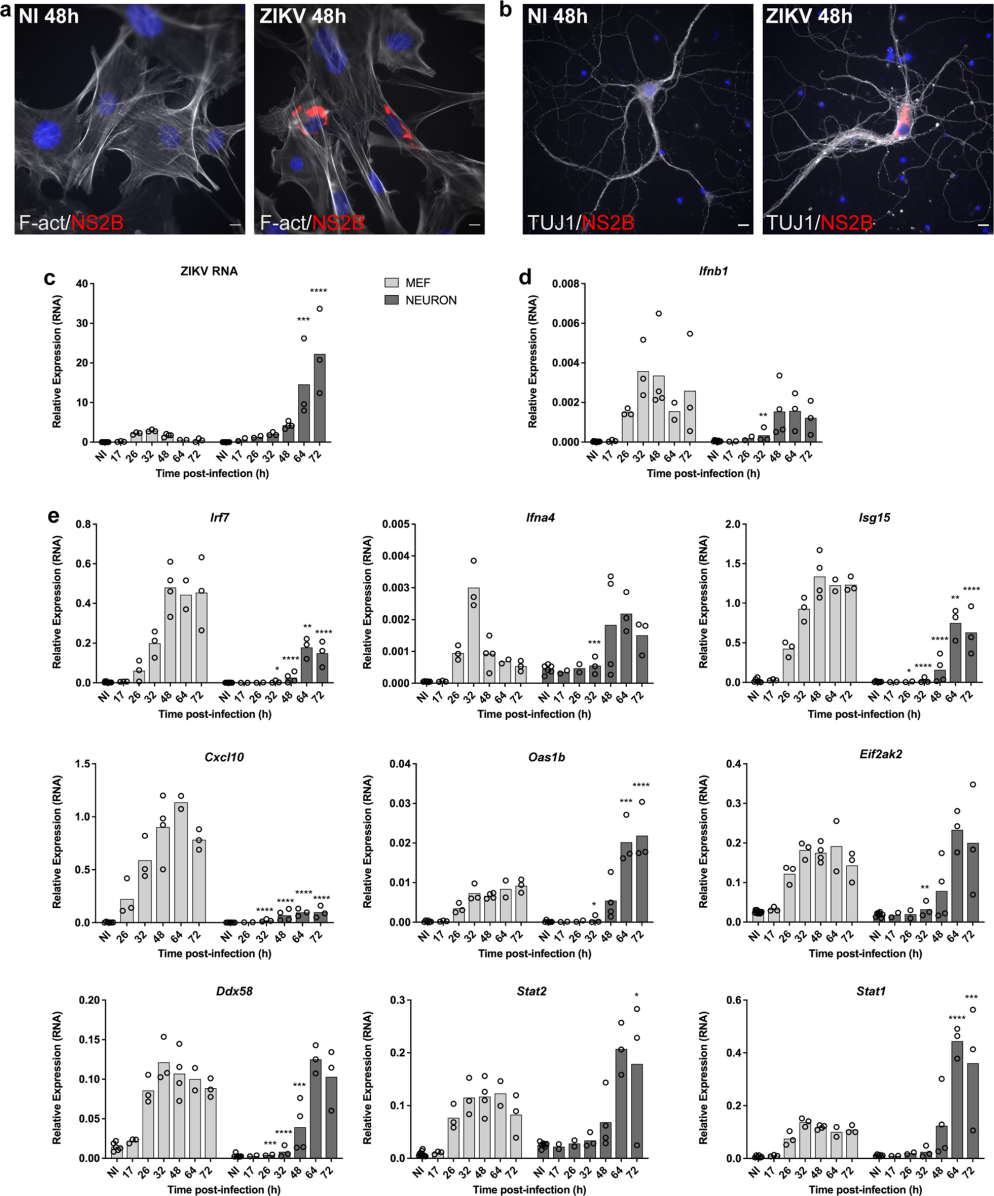
Primary cultured neurons (PCNs) from immunocompetent mice display a weak capacity to stop ZIKV replication. PCNs and MEFs from B6 embryos were either non-infected (NI) or ZIKV-infected at a multiplicity of infection (MOI) of 5. **a**, **b** Immunofluorescence using an anti-NS2B antibody (red) shows the presence of the non-structural NS2B protein encoded by ZIKV in **a** MEFs and **b** PCNs 48 h p.i. with filamentous actin (F-act) visualized in MEFs using phalloidin (grey) and neuronal beta-tubulin III visualized in PCNs using the TUJ1 antibody (grey). **c**–**e**) PCNs, unable to stop viral replication, display a delayed IFNB expression and response as shown by the relative expression levels of **c** ZIKV RNA and RNAs coding for **d** IFNB and **e** ISGs analyzed by RT-qPCR with respect to *Rplp0* used as reference gene. Symbols represent values obtained from *n* = 4 and 5 independent experiments for MEFs and PCNs respectively, with a minimum of two different times p.i. analyzed *per* experiment. Bars represent means. Significance between neurons and MEFs at the indicated times p.i. were assessed by two-way ANOVA and Sidak’s multiple comparison test. *P*-value < 0.0001 (****), < 0.001 (***), < 0.01 (**) and < 0.05 (*). Scale bars = 10 µm

Primary cultured neurons, microglia and MEFs grown on coverslips in 12 (neurons) and 24-well (microglia and MEFs) plates were fixed with 3.7% formaldehyde in PBS for 15 min and permeabilized with 1% Triton X-100 in PBS for 20 min. The cells were incubated for 1 h at room temperature with the corresponding primary antibodies diluted in PBS-5% bovine serum albumin. After washing with PBS the cells were next incubated for 45 min at room temperature with the corresponding secondary antibodies. In Fig. 8, samples were analyzed at room temperature with an inverted microscope (Leica DMI6000) equipped with a confocal Spining Disc scanning head (Spinning Disc Yokogowa CSU-X1M1). This system is equipped with a 63× lens, 1.4-numerical-aperture oil immersion lens (Plan APO) and motorized platine XY (*Märzhäuser Wetzlar* SCAN IM 127-83). Images were captured in the z-axis corresponding to the optical axis of the microscope at 0.30 μm intervals. MetaMorph 7.7.5 (Molecular devices) imaging software was used for image capture. The images were analyzed using the Image J software (National Institutes of Health). Maximum Intensity Projection and the Region of Interest (ROI) Manager Plugins of Image J were used to determine area of soma according to MAP2 labeling and quantify AT8 and AT100 content within soma. In Figs. 1 and 5, samples were analyzed using an inverted wide-field microscope (Leica DMI6000) with a 63x (oil) objective and a ORCA flash 4 camera (Hamamatsu). Z-series of images were taken at 2 µm increments. As previously, images were analyzed using ImageJ software (National Institutes of Health).

#### Brains slices

Immunofluorescence was performed at the confocal microscopy platform IMPRT (Institut de Médecine Prédictive et de Recherche Thérapeutique, Lille) as described previously [32]. Hippocampal sections from CC001 and CC071 mice (*n* = 3 for each mouse category) were acquired using LSM 710 confocal laser-scanning microscope (Carl Zeiss). The confocal microscope was equipped with a 488-nm Argon laser, 561-nm diode-pumped solid-state laser, and a 405-nm ultraviolet laser. The images were acquired using an oil 63× Plan-APOCHROMAT objective (1.4 NA). All recordings were performed using the appropriate sampling frequency (16 bits, 1024–1024 images, and a line average of 4).

### Western blot

Total protein extracts from primary cultured neurons and MEFs grown in 12-well plates were collected in 2× Laemmli buffer. After heating, protein extracts were loaded in NuPAGE 4–12% sodium dodecyl sulfate–polyacrylamide precast gels (Life Technologie). Loaded proteins were transferred on PolyVinyliDene diFluoride (PVDF) membrane. Membranes were incubated overnight at 4 °C with primary antibodies, washed with TBS-T (Tris Base Sodium Tween 0.1%) and incubated with the corresponding secondary antibodies for 1 h at room temperature. Imager ImageQuant LAS4000 was used for chemiluminescent protein detection. Relative quantification of proteins was carried out using Image J software.

### Statistics

Data was analyzed by two-way ANOVA and Sidak’s or Tukey’s multiple comparison tests, unpaired and paired Student’s *t* test or unpaired two-tailed Welsh’s test using Prism version 9.1 (GraphPad Software). The statistical analysis used and the number, *n*, of independent experiments is specified in the corresponding figure legends.

## Results

### A delayed induction of the IFNB response hampers the capacity of ZIKV-infected mature neurons from immunocompetent mice to control viral replication

In order to investigate the capacity of ZIKV-infected mature neurons to induce an efficient IFNB response, cortical PCNs prepared from C57BL/6J (B6) mouse embryos were infected with ZIKV. Murine embryonic fibroblasts (MEFs) prepared from the same embryos were used for comparison. The translation of new viral proteins was detected in both cell types as visualized at the single cell level using an antibody directed against the non-structural NS2B protein of ZIKV (Fig. 1a, b). RNAs from infected as well as non-infected (NI) PCNs and MEFs were collected at different times post-infection (p.i.) and the level of ZIKV RNA (Fig. 1c) as well as of mRNAs of genes coding for IFNB (Fig. 1d) and several ISGs (Fig. 1e) was analyzed by RT-qPCR.

ZIKV RNA was detected starting 17 h (h) p.i. in both cell types. In MEFs, the level of ZIKV RNA reached its maximum at 32 h p.i. and decreased thereafter, indicating a repression of viral replication (Fig. 1c). By contrast, the level of ZIKV RNA in PCNs that remained similar to that in MEFs until 32 h p.i., continued to increase thereafter and became significantly higher than in MEFs (Fig. 1c), indicating that PCNs were unable to control viral replication. Infectious viral particles were present in the supernatant of MEFs and PCNs cultures (Additional file 1: Fig. S1a) showing that infection was productive in both cell types. The kinetics of production of the new viral particles that accumulated in the corresponding supernatants throughout infection correlated with the kinetics of ZIKV RNA production, peaking at 48 h p.i. in MEFs, while in PCNs it kept increasing at least until 72 h p.i. (Additional file 1: Fig. S1a). However, this was not the case while comparing the total amount of viral particles with respect to the amount of RNA produced by each cell type. Notwithstanding higher ZIKV RNA levels produced by PCNs with respect to MEFs (Fig. 1c), PCNs produced overall less viral particles (Additional file 1: Fig. S1a), suggesting that ZIKV infection of PCNs could lead to the accumulation of viral RNA not incorporated into new viral particles.

Time-course analysis of the level of expression of RNAs coding for IFNB (Fig. 1d) showed that, compared with ZIKV-infected MEFs, ZIKV-infected PCNs displayed a delayed *Ifnb1* expression. While *Ifnb1* expression was induced in MEFs starting at 26 h p.i. and peaking at 32 h p.i., the induction of *Ifnb1* expression remained significantly lower in PCNs at least until 32 h p.i. (Fig. 1d). The delayed induction of *Ifnb1* expression in PCNs delayed the expression of all ISGs, resulting in significantly lower expression levels than in MEFs until 32–48 h p.i. (Fig. 1d). Yet, at late time points, the expression of some ISGs (*Oas1b*, *Stat1* and *Stat2*) increased beyond their peak expression in MEFs (Fig. 1e). The induction of a delayed IFNB expression and response by ZIKV-infected PCNs was not a general feature of PCNs since the infection of PCNs with the Newcastle Disease Virus (NDV) induced an early strong IFNB expression and response (Additional file 1: Fig. S1b).

**Fig. 2:**
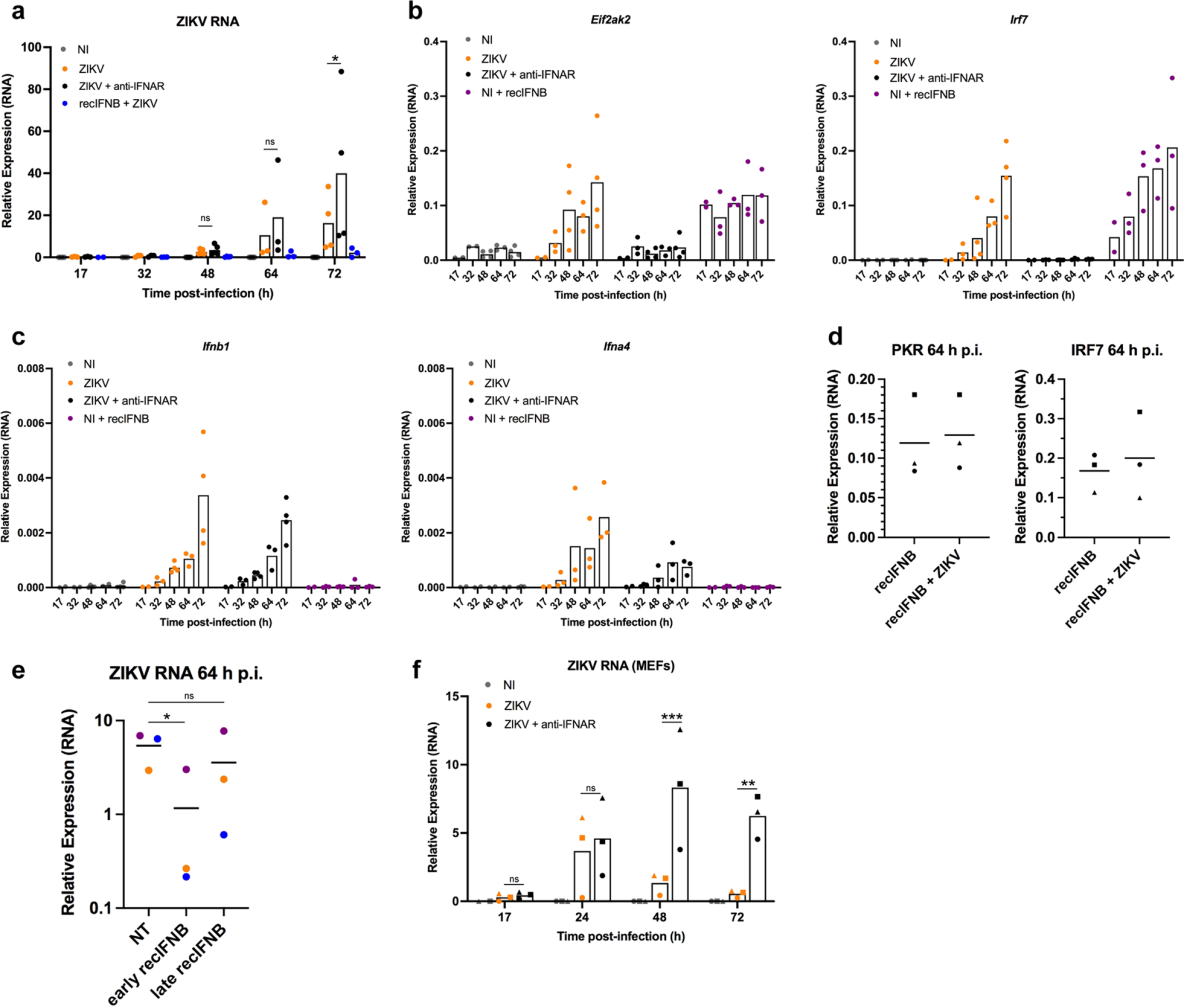
A delayed induction of IFNB response hampers the capacity of PCNs to stop ZIKV replication. PCNs from B6 embryos, non-infected (NI), non-treated (NT) or treated with anti-IFNAR antibodies or recIFNB before infection, were mock- or ZIKV-infected at a MOI of 5. **a**–**d** Neutralization of type-I IFN response with anti-IFNAR antibodies has no significant effect on ZIKV replication in PCNs until 72 h p.i. while treatment with recIFNB inhibits ZIKV replication from the onset of infection as shown by the relative expression of RNA levels analyzed by RT-qPCR with respect to *Rplp0* used as reference gene. Symbols represent values obtained from *n* = 5 independent experiments with varying conditions analyzed in each experiment with a minimum of two different times p.i. analyzed per condition per experiment. **a–c** Bars represent means without s.d. with significance assessed in **a** by two-way ANOVA and Tukey’s multiple comparisons test. **d** Means are represented in graph. **e** PCNs from B6 embryos ZIKV-infected at a MOI of 5, either non-treated (NT) or treated with recIFNB added before infection (early recIFNB) or 48 h p.i. (late recIFNB), were collected 64 h p.i.. Results correspond to those obtained from *n* = 3 independent PCNs shown in different colors. Means are represented in graph with significance assessed by paired t-test. Only early recIFNB significantly inhibited ZIKV RNA production measured by RT-qPCR with respect to *Rplp0* as reference gene. **f** MEFs from three different B6 embryos, either non-treated (NT) or treated with anti-IFNAR antibodies, were mock- or ZIKV-infected at a MOI of 5. Neutralization of type-I IFN response with anti-IFNAR antibodies significantly affected ZIKV starting at 48 h p.i. as shown by the relative expression of ZIKV RNA levels analyzed by RT-qPCR with respect to *Rplp0* used as reference gene. Symbols represent values obtained from each of the *n* = 3 different embryos. Bars represent means without s.d. with significance assessed in by two-way ANOVA and Tukey’s multiple comparisons test. *P*-value < 0.001 (***), < 0.01 (**), < 0.05 (*) and *ns* not significant

Because the IFNB response plays a major role in inhibiting viral replication, we hypothesized that the inability of PCNs to stop viral replication as compared to MEFs was the consequence of the delayed IFNB response observed following ZIKV infection in PCNs. In order to test this hypothesis, PCNs from B6 embryos were treated before and throughout ZIKV infection with either the MAR1-5A3 anti-IFNAR antibody or recombinant IFNB (recIFNB) (Fig. 2). RNAs were collected at different times p.i. as previously. By binding to the IFNAR receptor, MAR1-5A3 neutralizes the IFNB response, including IFNAs signaling, [33] therefore an increased level of viral RNA is expected following anti-IFNAR treatment if the IFNB response is indeed able to stop viral replication. Treatment of PCNs with MAR1-5A3 had no significant effect on the level of ZIKV RNA until 72 h p.i., indicating the absence of an effective IFNB response in ZIKV-infected PCNs at early times p.i. (Fig. 2a). As expected, MAR1-5A3 treatment neutralized the expression of the ISGs *Eif2ak2* and *Irf7* coding for PKR and IRF7 respectively at all time points (Fig. 2b). On the contrary, treating PCNs with recIFNB triggered an IFNB response (ISG expression) stronger than the one induced by ZIKV alone at early times p.i. (Fig. 2b) that was sufficient to inhibit ZIKV replication (Fig. 2a). As expected, treatment with either anti-IFNAR or recIFNB did not affect the level of the expression of the *Ifnb1* gene (Fig. 2c) that relies mainly on PRRs rather than IFNAR activation [19, 20]. Contrary to other ISGs, the expression of the genes coding for IFNAs (i.e., *Ifna4*) that depends on the presence of active, phosphorylated IRF7 for its transcription, requires both IFNB-induced IFNAR signaling necessary to induce the expression of IRF7 in many cell types (except (absent pDCs that constitutively express IRF7) and the activation of PRRs necessary to phosphorylate IRF7 [19]. Thus, as expected, treatment with recIFNB alone was not sufficient to induce *Ifna4* expression while MAR1-5A3 treatment inhibited the expression of *Ifna4* induced by ZIKV (Fig. 2c).

ZIKV infection of PCNs treated with recIFNB did not affect the capacity of recIFNB to induce ISG expression (Fig. 2d), indicating that the delayed induction of the IFNB response in ZIKV-infected PCNs is not a consequence of ZIKV affecting IFNAR signaling in this cell type. To further confirm the role of a delayed IFNB response in the inability of PCNs to stop ZIKV RNA replication, recIFNB was added at late times p.i. (48 h p.i.) and its effect was compared to that of recIFN added before infection as in Fig. 2a. While recIFNB added before infection (early recIFNB) significantly inhibited ZIKV RNA replication, this was not the case when recIFNB was added 48 h p.i. (Fig. 2e). Finally, treatment with anti-IFNAR inhibited the capacity of MEFs to stop ZIKV replication at earlier times p.i. (Fig. 2f) as compared to neurons (Fig. 2a), affecting the kinetics of ZIKV RNA replication in such a way that ZIKV-infected MEFs treated with anti-IFNAR reached 48 h p.i. a level of ZIKV expression equivalent to that reached by PCNs at 64 h p.i.

Overall, these results confirmed our hypothesis that the inability of ZIKV-infected PCNs to stop viral replication resulted from their inability to mount an efficient IFNB response early after infection as a consequence of a delayed induction of IFNB expression.

### Induction of the IFNB response is further delayed in PCNs from an immunocompetent mouse strain susceptible to ZIKV-induced disease

**Fig. 3:**
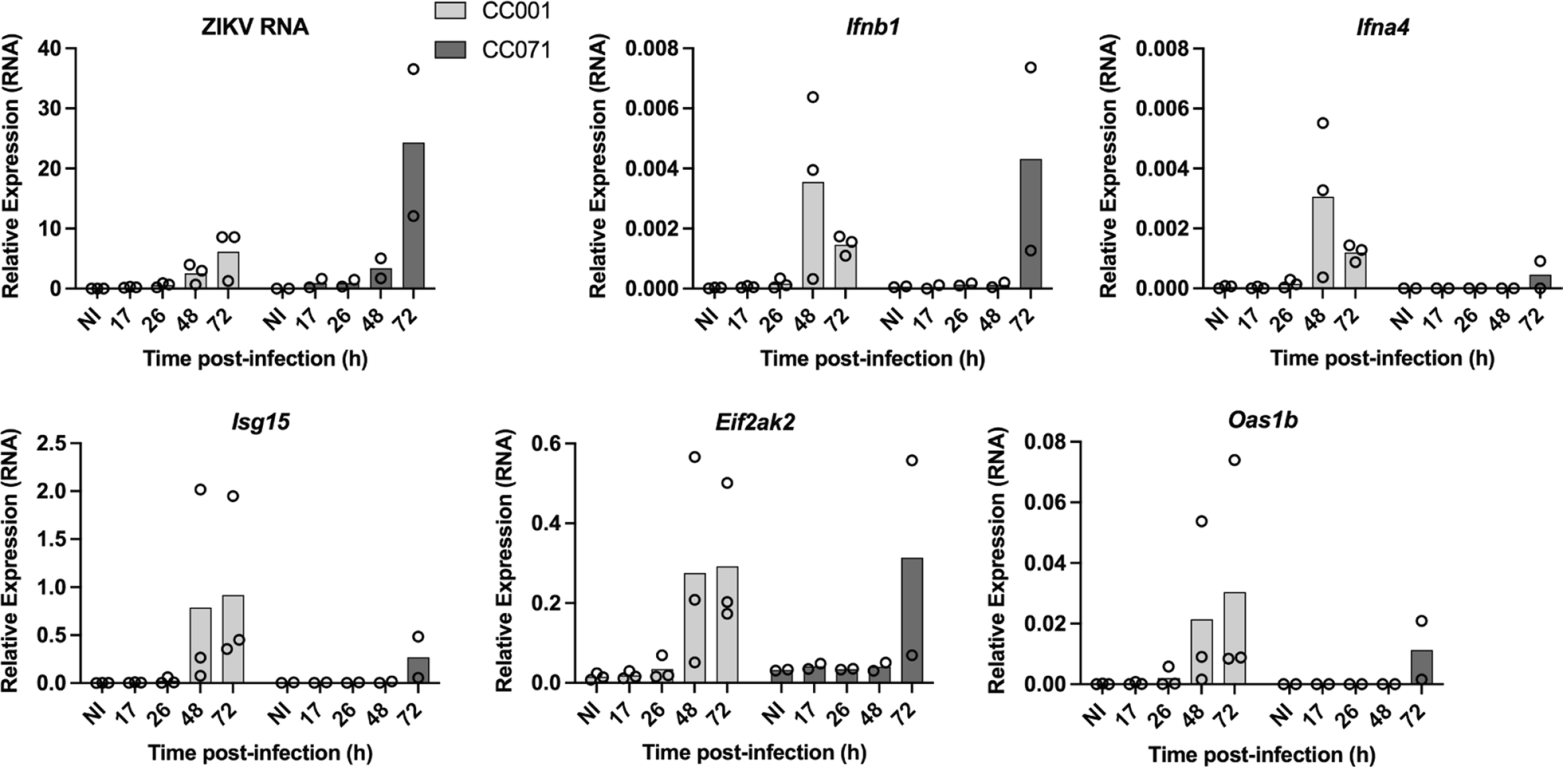
Susceptibility to ZIKV infection is correlated with the inability of PCNs to stop viral replication. PCNs from non-susceptible CC001 and susceptible CC071 immunocompetent mice were either non-infected (NI) or ZIKV-infected at a MOI of 5. Levels of ZIKV RNA and of RNAs coding for IFNB and ISGs were analyzed by RT-qPCR with respect to *Rplp0* used as reference gene. Symbols represent values obtained from *n* = 3 and 2 independent experiments for CC001 and CC071, respectively. Bars represent means

We have recently identified two mouse strains from the Collaborative Cross (CC) collection that exhibit contrasting susceptibility to ZIKV-induced disease after peripheral inoculation [25]. By contrast with the weakly permissive CC001 strain, the CC071 strain developed high brain viral load with histological signs of neuroinflammation after IC inoculation [25]. Of note, contrary to the high degree of mortality observed when CC071 mice were inoculated intraperitoneally after being treated with MAR1-5A3 [25], no mortality or clinical signs were observed up to 6 days post-infection after IC ZIKV inoculation in CC071 mice in the absence of MAR1-5A3. To test whether CC071 increased susceptibility was linked to a delayed neuronal *Ifnb1* expression and IFNB response, PCNs prepared from CC001 and CC071 embryos were infected with ZIKV and the RNA levels of ZIKV, IFNB and ISGs were analyzed at different times p.i. as previously (Fig. 3). While ZIKV replicated in PCNs from CC001 mice similarly to B6 PCNs, CC071 PCNs were even less capable of stopping ZIKV replication than CC001 PCNs with a further delayed induction of *Ifnb1* expression and IFNB response (Fig. 3).

These results highlight the link between the susceptibility of adult immunocompetent mice to ZIKV-induced disease and the inability of mature PCNs derived from these mice to set up an early IFNB expression and response in order to efficiently stop ZIKV replication.

### In vivo, CC071 mice display a strong ZIKV-induced microglia and pro-inflammatory response activation in the absence of microglial cell infection

Microglia activation has been previously reported as the main histological feature observed after IC ZIKV infection of adult mice [14, 18, 25]. However, in human as well as mouse adult brain tissue neurons but not microglial cells were identified as infected by ZIKV [14, 18]. We have previously published immunohistochemistry results suggesting that higher levels of microglia activation were induced in the brain of CC071 as compared to CC001 mice following ZIKV IC inoculation [25]. The capacity of ZIKV to activate microglia of CC071 *versus* CC001 mice was further analyzed in this work on the one hand, with respect to the capacity of ZIKV to induce brain IFNs-I and pro-inflammatory responses and on the other hand, with respect to the capacity of ZIKV to infect or not infect CC071 microglial cells. For this, adult non-susceptible CC001 and susceptible CC071 mice were inoculated IC with ZIKV or PBS and necropsied 6 days p.i. (d.p.i.). One brain hemisphere was used for gene expression analysis and the other for immunofluorescence coupled to confocal microscopy analysis. In agreement with previously published results [25], at this time point after infection, significantly higher levels of ZIKV RNA (Additional file 2: Fig. S2a) and Iba1 (a specific marker of microglial cells) labeling (Additional file 2: Fig. S2b) were observed in brain samples from ZIKV-infected CC071 compared to PBS-treated or ZIKV-infected CC001 mice. In ZIKV-infected CC071 brain samples, Iba1 expressing microglia that intermingled with NeuN+ neurons displayed hypertrophic cell bodies and short asymmetric processes (Additional file 2: Fig. S2c), a morphology characteristic of active microglia [34, 35].

**Fig. 4:**
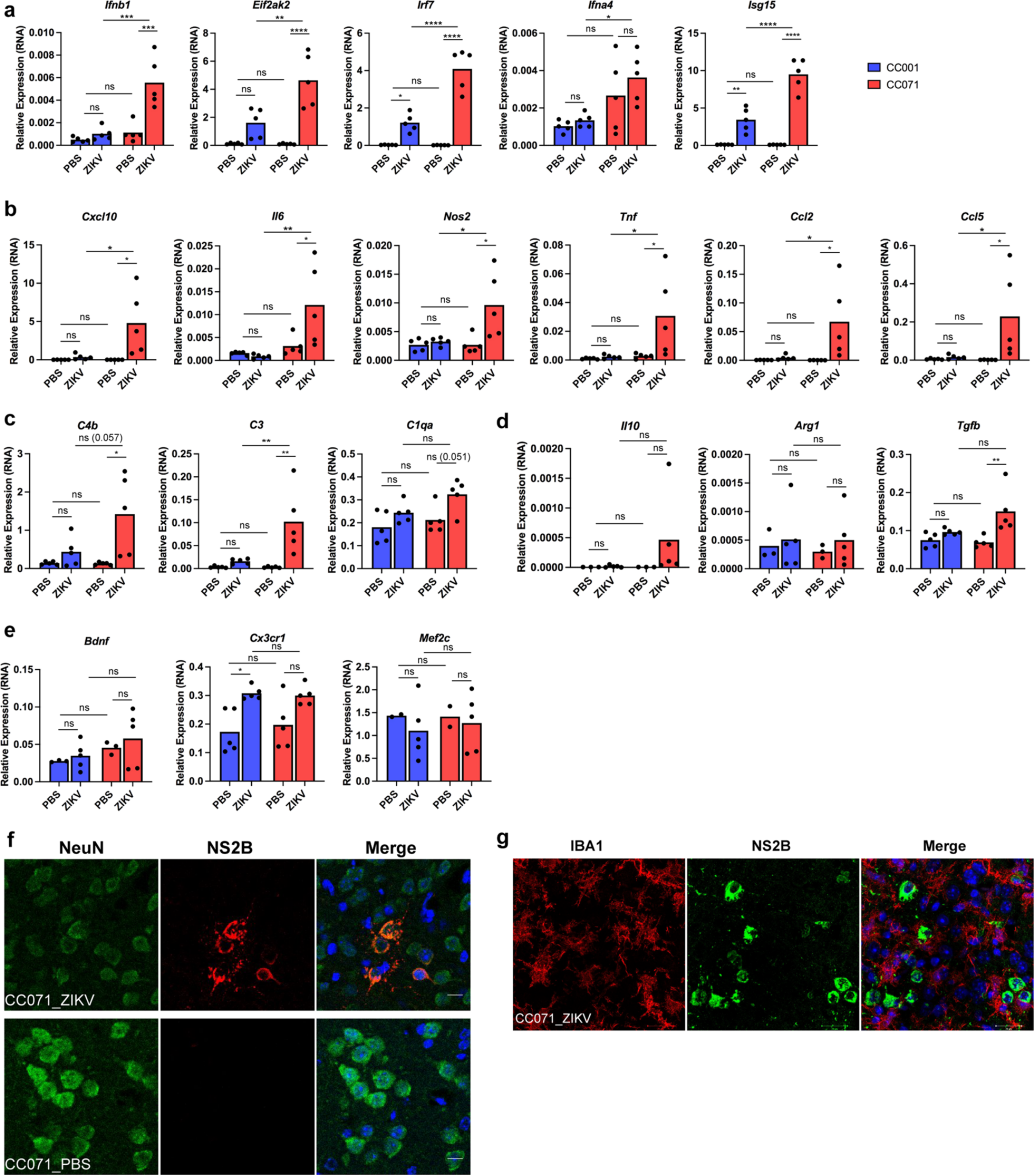
ZIKV-infection induces the activation of microglia and of IFNs-I and pro-inflammatory responses in the brain of immunocompetent CC071 mice, in the absence of microglia infection. CC001 and CC071 mice (5–6 week-old) were necropsied at day 6 following IC inoculation of either PBS or 10^5^ FFU of ZIKV. **a**–**e** As compared to non-susceptible CC001 mice, ZIKV infection of susceptible CC071 mice induced a significantly higher expression of mRNAs coding **a** for IFNB and ISGs and for factors associated with the **b** the pro-inflammatory response and **c** the complement cascade without significantly affecting genes associated with **d** the anti-inflammatory response and **e** the homeostatic “off” state of microglia as determined by RT-qPCR analysis of total brain extracts with respect to *Hrpt1* used as reference gene. Symbols represent individual mice. Data from *n* = 5 CC001 and CC071 mice respectively are means without s.d. with significance assessed by two-way ANOVA Tukey’s multiple comparisons test. *P*-value < 0.0001 (****), < 0.001 (***), < 0.01 (**), < 0.05 (*) and *ns* not significant; *P*-values comprised between 0.05 and 0.1 (considered as near significant) are indicated. **f**, **g** ZIKV induced microglia activation in CC071 mice brains while only infecting neurons as determined by immunofluorescence and confocal microscopy with neurons labeled with anti-NeuN (green), microglial cells with anti-Iba1 antibody (red), ZIKV-infected cells with anti-NS2B antibody (red or green) and DNA labeled with DAPI (blue). Single confocal sections (z projection) and the corresponding merge images are shown. Scale bars in **f** 10 µm and **g** 20 µm

Gene expression analysis of brain extracts of ZIKV-infected susceptible CC071 mice revealed that microglia activation was associated not only with a stronger *Ifnb1* and ISGs expression (Fig. 4a) but also with a robust pro-inflammatory response by comparison with ZIKV-infected CC001 and PBS-treated mice (Fig. 4b). In particular, significant (or near significant) higher levels of expression were detected for genes coding for main pro-inflammatory cytokines and chemokines (*Cxcl10*, *Il6*, *Tnfa*, *Ccl2*, *Ccl5*) and the *Nos2* gene coding for iNOS, the enzyme responsible of the production of nitric oxide (Fig. 4b) as well as for genes coding for complement factors (*C4b*, *C3* and *C1qa*; Fig. 4c), which are all characteristic of the active state of microglia and often associated with neurotoxic consequences [26, 27, 36]. On the contrary, no significant differences were observed between ZIKV-infected CC071 and CC001 mice in the expression levels of genes related with an anti-inflammatory (*Il10*, *Arg1*, *Tgfb*; Fig. 4d) and the homeostatic and neuroprotective “off” state of microglia (*Bdnf*, *Cx3crl* and *Mef2c*; Fig. 4e). For all the genes associated with the IFNB response (Fig. 4a), the pro-inflammatory response and the complement cascade (Fig. 4b and c) that displayed significant or near significant different expression levels in brain extracts from ZIKV-infected CC071 as compared to ZIKV-infected CC001 mice, no significant differences were observed between the two strains in the absence of infection (PBS treated mice).

Immunofluorescence labeling using an antibody directed against the ZIKV NS2B protein identified viral infection predominantly in NeuN+ neurons of CC071 mice (Fig. 4f). By contrast, no infection was detected in microglial cells labeled with anti-Iba1 antibodies even in those located close to ZIKV-infected (NS2B+) neurons (Fig. 4g).

In conclusion, ZIKV infection induced microglia activation in the brain of ZIKV-infected CC071 mice susceptible to ZIKV-induced disease in correlation with higher levels of induction of the IFNB and pro-inflammatory responses as compared to PBS-treated and CC001-infected mice less susceptible to ZIKV infection, even though microglial cells from CC0071 mice were seemingly not infected by ZIKV.

### Type I IFNs secreted by ZIKV-infected neurons activate non-infected microglial cells

**Fig. 5:**
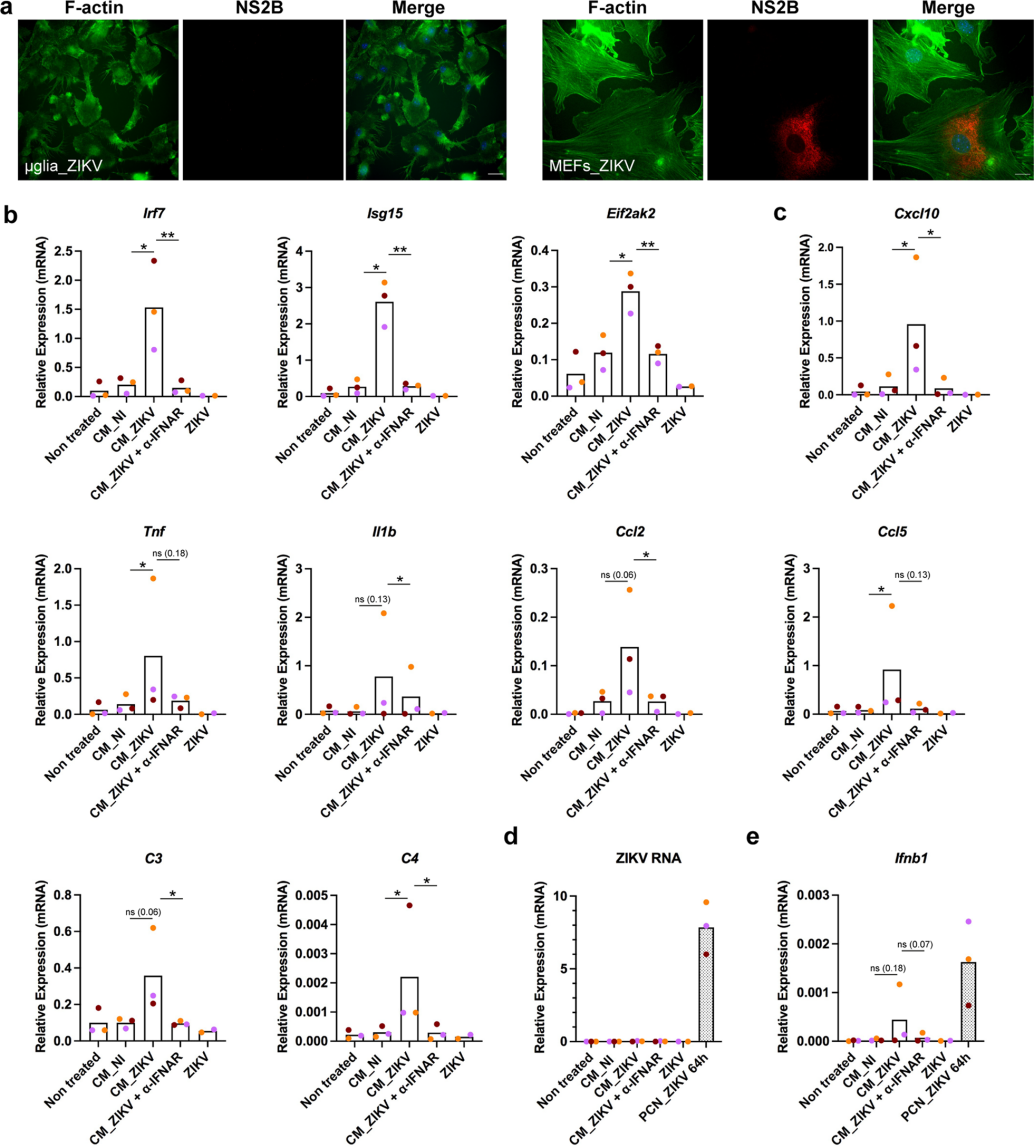
Type I IFNs present in conditioned medium from ZIKV-infected neurons activate non-infected microglial cells. ZIKV does not infect primary cultured microglial cells as determined by **a** immunofluorescence of MEFs and microglial cells from B6 mice exposed ZIKV at a MOI of 5 and labeled 24 h p.i. with an anti-NS2B antibody (red) along with staining of filamentous actin (F-actin) with phalloidin (green). Scale bars = 10 µm. Conditioned medium from ZIKV-infected PCNs induced the activation of the expression level of genes coding for IFNB (*Ifnb1*), ISGs (*Irf7*, *Isg15*, *Eif2ak2*) and factors associated with the pro-inflammatory “on” state of microglia cells (*Cxcl10*, *Tnf*, *Il1b*, *Ccl2*, *Ccl5*, *C3*, *C4*) through IFNs-I signaling. **b**–**e** Primary cultured microglial cells from B6 mice were treated with conditioned media recovered at 64 h p.i. from *n* = 3 B6 PCNs either non-infected (CM_NI) or ZIKV-infected (CM_ZIKV) as in Fig. 1, in the presence or absence of anti-IFNAR antibodies (α-IFNAR). As a control, microglial cells were treated with the same amount of ZIKV as the one present in CM_ZIKV. Results correspond to those obtained from three independent primary culture of microglia (with symbols corresponding to each independent culture shown in different colors) each one treated with CM from one of the three independent PCNs. RNA levels were determined by RT-qPCR with respect to *Rplp0* used as reference gene. The levels of ZIKV and IFNB RNA present in the three ZIKV-infected PCNs from which the CMs used here were collected (grey bars) are shown in **d** and **e** respectively. Bars represent means without s.d. with significance assessed by ratio paired *t*-test. *P*-value < 0.01 (**), < 0.05 (*) and *ns* not significant; *p*-values near significance are indicated

The inability of ZIKV to infect microglial cells was further confirmed in vitro by incubating primary cultured microglial cells prepared from B6 newborn mice with ZIKV at an MOI = 5 during 24 or 48 h before fixation and labeling with anti-NS2B antibodies (Fig. 5a). No NS2B+ microglial cells were observed while over 10% of B6 MEFs infected in parallel were NS2B+ (Fig. 5a).

In order to investigate the capacity of factors secreted by ZIKV-infected neurons (among them IFNs-I) to activate microglia, primary cultured microglia prepared from newborn B6 mice were incubated with the conditioned media (CM) collected from non-infected (CM_NI) or ZIKV-infected (CM_ZIKV) B6 PCNs analyzed as in Fig. 1, in the presence or absence of anti-IFNAR antibodies. Three independent primary cultures of microglia were incubated with conditioned media collected at 64 h p.i from three independent ZIKV-infected PCNs experiments (corresponding to grey columns in Fig. 5d and e). In parallel, microglial cells were incubated with the same amount of ZIKV present in the CM_ZIKV as determined by titration. Gene expression analysis carried out 6 h post-treatment showed that CM from ZIKV-infected neurons induced the activation of the expression of not only ISGs (*Irf7*, *Isg15, Eif2ak2*; Fig. 5b) but also of genes associated with the pro-inflammatory “on” state of microglia (*Cxcl10*, *Tnf*, *Il1b*, *Ccl2*, *Ccl5*, *C3*, *C4*; Fig. 5c). The expression level of *Tnf* and *C3* genes reached by microglial cells 6 h after treatment with CM_ZIKV (from 0.19 to 1.8 and 0.2 to 0.6, respectively) was respectively over 10^3^ and 10^2^ times higher than the expression level measured for these genes in ZIKV-infected PCNs 64 h p.i. (0.00014 and 0.001, respectively). For all the genes tested, the induction of expression by CM_ZIKV was significantly or near significantly reduced in presence of anti-IFNAR antibodies (Fig. 5c) showing that this induction was strongly dependent on IFNs-I signaling. No induction of expression was observed in the presence of ZIKV alone, ruling out a direct effect of the virus on microglial cells. In agreement with the inability of ZIKV to infect microglial cells, no ZIKV RNA was detected after treatment with either ZIKV alone or CM_ZIKV, while high levels of viral RNA were found in ZIKV-infected PCNs from which CMs were collected (Fig. 5d). Interestingly, conditioned medium from ZIKV-infected neurons was also capable to induce some level of expression of IFNB RNA itself through a mechanism dependent upon IFNAR signaling, independent of virus infection (Fig. 5e). In the case of one of the three independent primary microglial cultures analyzed, microglial cells reached 6 h post-treatment a level of *Ifnb1* expression close to that reached by ZIKV-infected PCNs 64 h p.i. (Fig. 5e).

These results demonstrate that IFNs-I secreted by ZIKV-infected PCNs are capable to directly activate non-infected primary cultured microglial cells.

### In vivo, ZIKV infection induces a DAM-like phenotype in the brain of CC071 mice susceptible to ZIKV-induced disease

**Fig. 6:**
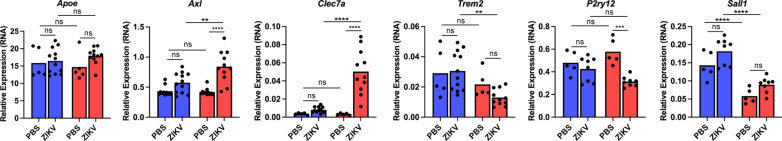
ZIKV-infection induces a DAM-like phenotype in the brain of immunocompetent CC071 mice susceptible to infection. CC001 and CC071 mice (5–6 week-old) were necropsied at day 6 following IC inoculation of either PBS or 10^5^ FFU of ZIKV. In the brain of CC071 as compared to CC001 mice, ZIKV affects the level of RNA expression of genes associated with a DAM phenotype as determined by RT-qPCR analysis of total brain extracts from ZIKV-infected CC001 and CC071 mice with respect to *Hrpt1* used as reference gene. Symbols represent individual mice. Data from *n* = 5 CC001 and CC071 mice respectively are means without s.d. with significance assessed by two-way ANOVA and Tukey’s multiple comparisons test. *P*-value < 0.0001 (****), < 0.001 (***), < 0.01 (**) and *ns* not significant

A role for IFNs-I in activating microglial cells has been described in vivo in the brain of AD-like mouse models [37]. In this case, microglial cells displayed a gene expression pattern close to the transcriptional signature of DAM or microglial neurodegenerative (MGnD) phenotype characterized in association with the development of various neurodegenerative diseases [27, 38]. In order to analyze the capacity of ZIKV to induce a DAM-like phenotype, we measured the expression level of some of the major genes (other than the DAM-associated genes related to the pro-inflammatory response and the complement cascade measured in Fig. 4) specifically either up-regulated (such as *Apoe*, *Axl* and *Clec7a*) or down-regulated (such as *Sall1* and *P2ry12*) in DAM or MGnD phenotype [26, 27] such as *Apoe*, *Axl* and *Clec7a* (up-regulated in DAM) and *Sall1* and *P2ry12* (down-regulated in DAM). As shown in Fig. 6, *Axl* and *Clec7a* were significantly up-regulated in response to ZIKV infection in the brain of CC071 as compared to CC001 mice while *P2ry12* was significantly down-regulated (Fig. 6). The basal level of expression of the *Sall1* gene was significantly lower in CC071 as compared to CC001 mice and remained so after ZIKV infection (Fig. 6). As for the *Trem2* gene, a regulator of inflammation in microglia, that is up-regulated at later stage 2 of DAM [27, 39] but down-regulated at early stages of AD [40], was found significantly down-regulated at 6 d.p.i. in ZIKV-infected CC071 as compared to CC001 brain (Fig. 6).

Overall, these results indicated that at 6 d.p.i., microglial cells from ZIKV-infected susceptible mice display a phenotype suggestive of a stage 1 DAM-like phenotype.

### ZIKV-infected mice develop a pathological phosphorylation of Tau

The DAM gene expression profile has been found closely linked to AD development and the pathological phosphorylation of Tau protein (pTau) [40, 41]. In order to explore the eventual correlation between ZIKV-induced DAM and pTau, the presence of pTau in the brain of PBS-treated and ZIKV-infected CC071 and CC001 mice was analyzed at 6 d.p.i. by immunofluorescence and confocal microscopy using the AT8, AT100 and AD2 Tau phosphorylation-dependent antibodies that specifically recognizes respectively the PSer202-Thr205 Tau epitope (AT8), PThr212-Ser214Tau epitope (AT100) and PSer396-Ser404 epitope (AD2) which are hallmarks of AD and other tauopathies [28, 30, 42].

**Fig. 7:**
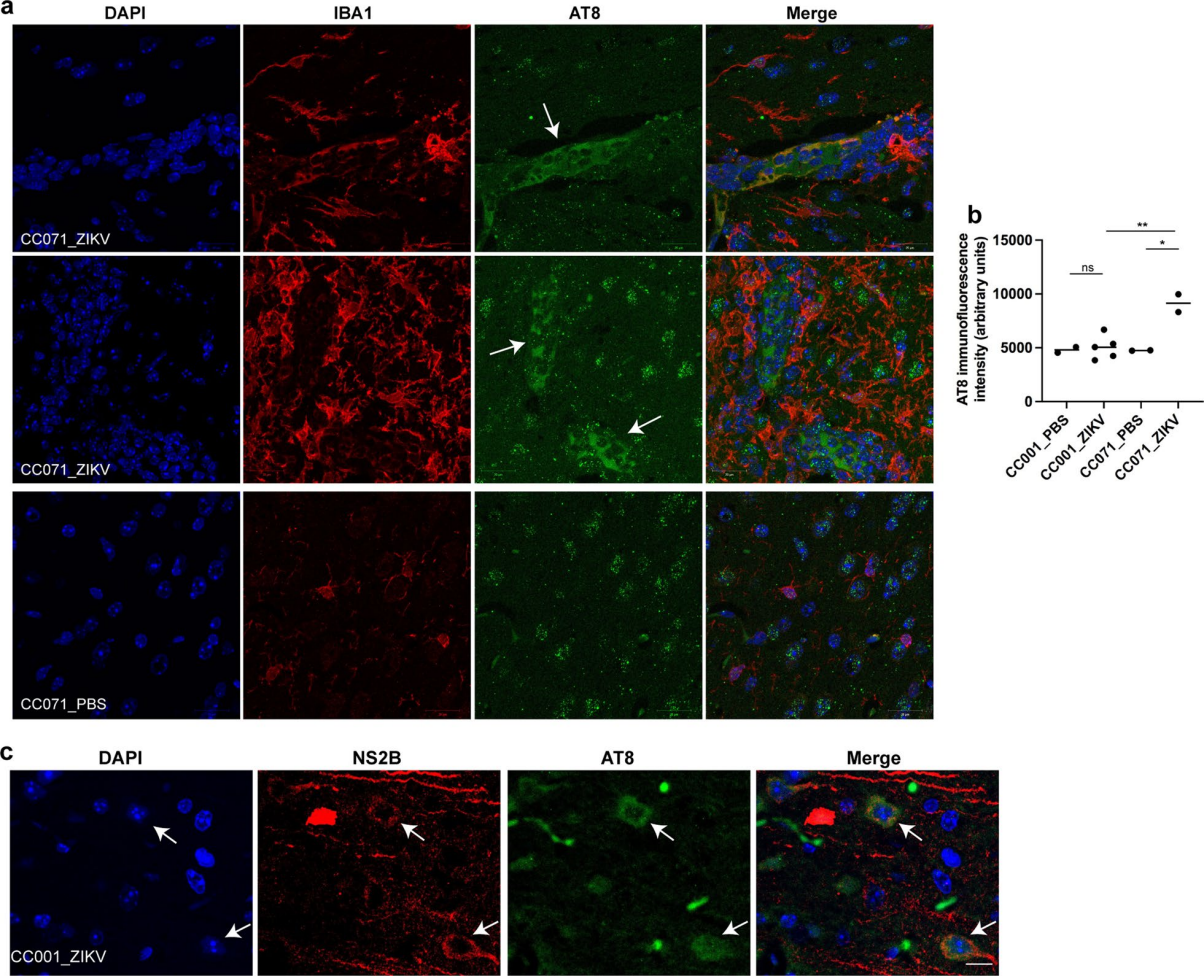
ZIKV infection induces pathological Tau phosphorylation in vivo. CC001 and CC071 mice (5–6 week-old) were necropsied at day 6 following IC inoculation of either PBS or 10^5^ FFU of ZIKV. In vivo, ZIKV infection induced pTau recognized by AT8 antibodies predominantly in the brain of CC071 mice as determined by immunofluorescence and confocal microscopy with **a**, **c** nuclear DNA labeled with DAPI (blue), pTau labeled with the AT8 antibody (green), **a** microglial cells labeled with anti-Iba1 antibody (red) and **c** ZIKV-infected neurons labeled with anti-NS2B antibody (red). Arrows indicate in **a** clusters of pTau positive CC071 neurons surrounded by active microglial cells and in **c** ZIKV-infected pTau positive CC001 neurons. Single confocal sections (z projection) and the corresponding merge images are shown. Scale bars = 20 µm. **b** AT8 immunofluorescence intensity was quantified in cells from CC001 PBS-treated (*n* = 2 brains, 42 cells), CC001 ZIKV-infected (*n* = 5 brains, 107 cells), CC071 PBS-treated (*n* = 2 brains, 41 cells) and CC071 ZIKV-infected (*n* = 2 brains, 277 cells) mice. Symbols represent average values obtained for each brain with significance assessed by unpaired *t*-test. Means are indicated in graph. *P*-value < 0.01 (**), < 0.05 (*) and *ns* not significant

Clusters of neurons displaying cytoplasmic AT8 labeling were observed at the frontier of the cortex and the hippocampus, in the brain of ZIKV-infected but not PBS-treated CC071 mice (Fig. 7a). Significant increase of AT8 labeling, quantified in Fig. 7b, was observed in 2 of the 5 brains analyzed. These clusters appeared surrounded by active microglial cells displaying strong Iba1 labeling compared to PBS-treated CC071 mice (Fig. 7a). Under these conditions, no increase of AT8 labeling was observed in the brain of ZIKV-infected as compared to PBS-treated non-susceptible CC001 mice (Additional file 3: Fig. S3) except in some rare regions of the cortex where cells expressing somatic labeling of ZIKV NS2B protein were also more strongly labeled with the AT8 antibody (Fig. 7c). Contrary to the increased of cytoplasmic AT8 labeling observed in CC071 mouse brains at 6 d.p.i., ZIKV infection did not trigger cytoplasmic AT100 and AD2 labeling neither in CC071 nor in CC001 mouse brains at this time p.i. (Additional file 4: Fig. S4).

Overall, these results demonstrate that ZIKV infection can rapidly (6 days p.i.) induce pathological Tau phosphorylation in vivo in association with ZIKV replication and microglia activation.

### ZIKV infection induces pathological phosphorylation of Tau protein independently of glial cells

**Fig. 8:**
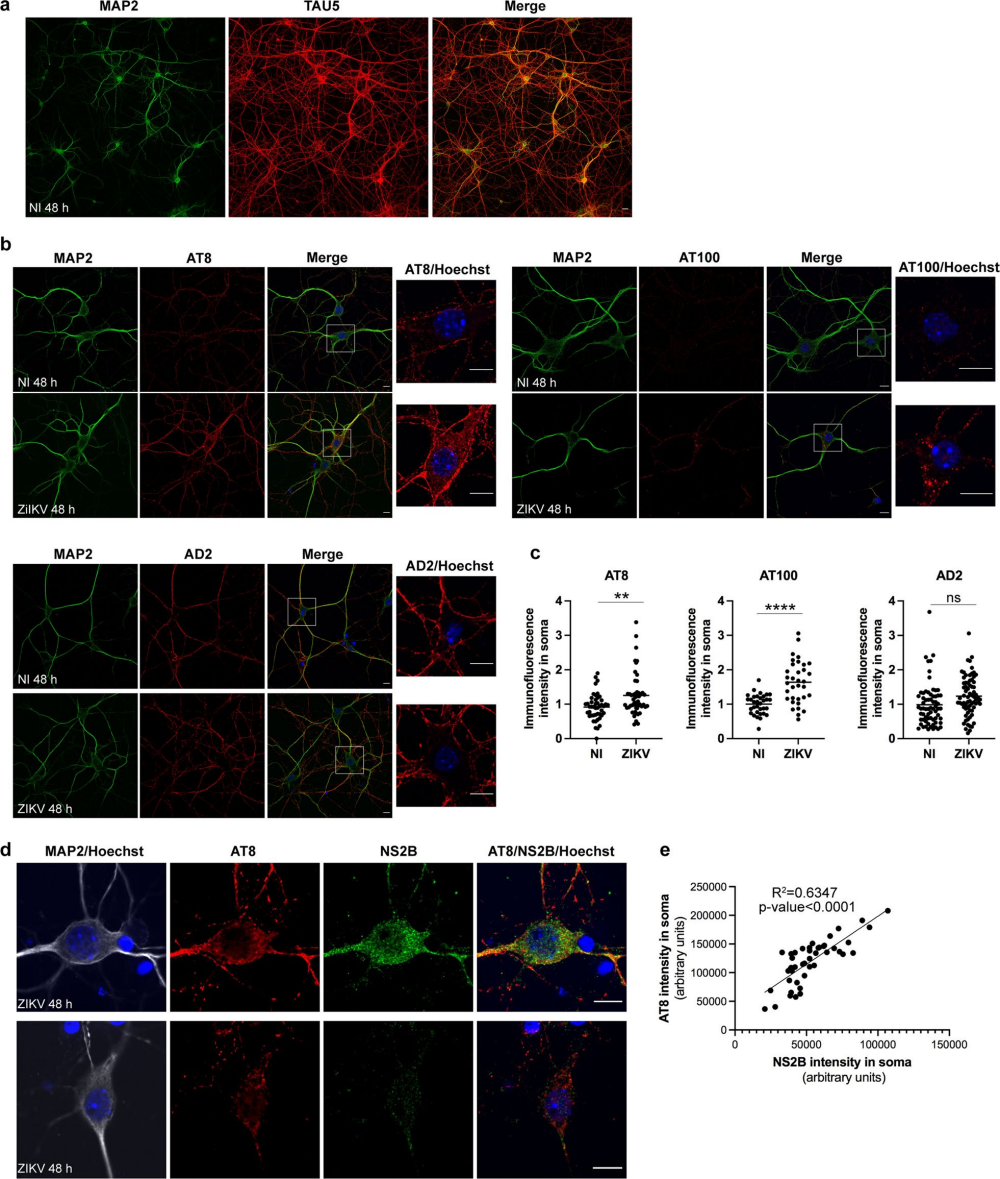
In vitro, ZIKV infection induces pTau in the absence of glial cells. PCNs from B6 embryos were either non-infected (NI) or ZIKV-infected (ZIKV) at a MOI = 5. ZIKV infection of PCNs induces pTau recognized by AT8 and AT100 antibodies 48 h p.i. predominantly in the soma of PCNs as determined by immunofluorescence and confocal microscopy with **a**, **b** nuclear DNA labeled with Hoechst (blue), the somatodendritic MAP2 protein with anti-MAP2 antibody (green), total Tau with Tau5 antibody (red) and pTau with AT8, AT100 and AD2 antibodies (red). In **c** the immunofluorescence intensity of pTau labeling with AT8, AT100 and AD2 antibodies in soma was quantified using ImageJ with the surface of soma determined according to MAP2 labeling while excluding dendrites. Symbols represent values normalized to NI obtained for each single cell. For AT8, *n* = 3 independent experiments for PBS and ZIKV conditions with 33 and 34 cells analyzed, respectively. For AT100, *n* = 3 independent experiments for PBS and ZIKV conditions with 48 and 50 cells analyzed, respectively. For AD2, *n* = 2 independent experiments for PBS and ZIKV conditions with 48 and 52 cells analyzed, respectively. Means are shown in graph with significance assessed by unpaired two-tailed Welch’s *t* test. *P*-value < 0.0001 (****), < 0.01 (**) and *ns* not significant. Among ZIKV-infected cells, the intensity of AT8 labeling in soma was positively correlated with the intensity of NS2B labeling as determined in **d** by immunofluorescence and confocal microscopy with nuclear DNA labeled with Hoechst (blue), the somatodendritic MAP2 protein with anti-MAP2 antibody (grey), pTau with the AT8 antibody (red) and the NS2B protein encoded by ZIKV with anti-NS2B antibodies (green). In **e** the immunofluorescence intensity of pTau and NS2B labeling in soma was quantified using ImageJ with the surface of soma determined according to MAP2 labeling while excluding dendrites. Symbols represent raw values obtained for each single cell from *n* = 2 independent experiment with the square of the sample Pearson correlation coefficient (R^2^) indicated. **a**, **b**, **d** Representative single confocal sections and the corresponding merge images are shown. Scale bars = 10 µm

In order to examine the capacity of ZIKV to directly induce neuronal Tau phosphorylation in the absence of glial cells, non- and ZIKV-infected PCNs analyzed in Fig. 1 for RNA expression levels were fixed at 48 h p.i. and incubated with the Tau phosphorylation-dependent antibodies AT8, AT100 and AD2 as described above for mouse brains [28, 30, 42]. We also used an antibody directed against total Tau protein (Tau5) for which an axonal distribution is expected in the case of mature neurons and an antibody directed against the microtubule associated protein 2 (MAP2) that is exclusively present in neuron cell bodies and dendrites [43]. Total Tau protein labeled with Tau5 antibody was predominantly observed in axons not co-localizing with MAP2 (Fig. 8a) indicative of neuron maturation, while also present in the cell bodies and to a lesser extent in dendrites. Representative confocal microscopy images shown in Fig. 8b indicated that ZIKV infection induced an increase of the pathological phosphorylated forms of Tau recognized by AT8 and AT100 antibodies but not of the form recognized by the AD2 antibody (Fig. 8b). The enhancement of AT8 and AT100 labeling was predominantly observed in the cell body (Fig. 8b, insets). The quantification of AT8 and AT100 intensity in soma of non- and ZIKV-infected PCNs from three independent experiments confirmed a significant increase of these two pathological phosphorylated forms of Tau protein following ZIKV infection of PCNs (Fig. 8c). In order to determine if the increase of pTau among ZIKV-infected PCNs was correlated with the degree of infection, as suggested by in vivo results obtained with CC071 and CC001 mice, ZIKV-infected PCNs were co-labeled with AT8 and the anti-NS2B antibody. Forty-eight hours after ZIKV infection, the neurons that expressed NS2B were also identified as those that displayed the highest amount of AT8 labeling (Fig. 8d). The blind quantification of the intensity of AT8 and NS2B labeling in soma of ZIKV-infected PCNs showed a highly significant positive correlation between NS2B and AT8 labeling (Fig. 8e).

Overall these results indicate that ZIKV infection has the capacity to directly induce pathological Tau phosphorylation in neurons in the absence of microglia or other glial cells with the degree of ZIKV-induced pTau being directly correlated with the degree of neuronal ZIKV infection.

## Discussion

Immediately following infection, a race starts in primo infected cells between virus replication and the cellular innate antiviral response. The result of this race is crucial for the outcome of the infection, deciding between either viral clearance and control of the infection or virion production and infection propagation. We show here that, as a result of a delayed induction of IFNB expression and response, infected neurons from immunocompetent mice lose the race against ZIKV, a trait that was further increased in neurons from CC071 mice susceptible to ZIKV-induced disease. The inability of mature neurons, unlike MEFs, to stop ZIKV replication is representative of the higher susceptibility of the CNS to ZIKV infection as compared to peripheral tissue observed in adult immunocompetent mice [18]. Neurons being unable to stop viral replication, ZIKV RNA persisted at high levels in PCNs and brain of ZIKV-infected CC071 mice at least 3 and 6 d.p.i. respectively, which is in accordance with the persistence of replicating ZIKV that has been observed in the brain of immunocompetent adult B6 mice [44].

Alongside with an enhanced expression of ZIKV RNA, we observed in vivo, in the brain of CC071 immunocompetent mice 6 d.p.i., an increased level of active Iba1 expressing microglial cells occasionally engulfing neurons, which is in agreement with results previously obtained by other groups describing high levels of microglia activation following ZIKV infection of adult mice brain [14, 18, 25, 44]. Gene expression analysis of brain extracts of ZIKV-infected susceptible mice allowed the identification of a gene expression profile close to the molecular signature of disease associated microglia (DAM) described in AD [26, 27]. This signature is characterized by the up-regulation of pro-inflammatory genes associated with neurotoxic functions such as *Il6*, *Tnf*, *Ccl2*, *Cxcl10* and the combination of up- and down-regulation of specific genes such as *Axl* and *Clec7a* (up-regulated in DAM as well as in ZIKV-infected CC071 brain extracts) and *P2ry12* (down-regulated in DAM as well as in ZIKV-infected CC071 brain extracts). *Axl* positively regulates the phagocytic activity of microglia in association with the establishment of an inflammatory environment [45] and *Sall1* inactivation converts resting microglia into active inflammatory phagocytes [46]. Thus, the up-regulation of *Axl* alongside with the constitutive low level of *Sall1* gene expression in CC071 mice, constitute a favorable ground for the development of a pro-inflammatory DAM-like phenotype in the brain of ZIKV-infected mice.

However, in agreement with results previously published by other groups [14, 18, 44], microglial cells were not identified as infected by ZIKV even in the case of CC071 mice susceptible to ZIKV-induced disease raising the question of the mechanisms underlying microglia activation following brain ZIKV infection. Treatment of primary cultured microglia with conditioned medium from ZIKV-infected PCNs demonstrated that IFNs-I secreted by infected neurons were capable to induce, in the absence of infection and ZIKV replication, a microglial expression profile similar to the one observed in brain extracts from ZIKV-infected CC071 mice susceptible to ZIKV-induced disease. These results demonstrated that IFNs-I had the capacity to directly activate microglia as it had been previously suggested in the context of AD [37] and in relation with aging [39].

A role for Zika virus-induced TNFa-signaling in the induction of psychiatric disorders has been recently reported [47]. In this case, authors suggested that ZIKV-infected neurons would be the main source of TNFa expression. However, we show here that the level of *Tnf* expression reached by microglial cells 6 h after treatment with conditioned media from ZIKV-infected neurons was 10^3^ times superior to the level reached by ZIKV-infected neurons 64 h p.i., suggesting that microglial cells would contribute to a much larger extent than neurons to the level of *Tnf* expression in the brain of ZIKV-infected mice. Under these conditions, microglial cells also displayed a high level of expression of the gene coding for complement C3 that directly affects the integrity and function of neurons in relation with the establishment of cognitive disorders [48, 49] and the development of Tau pathology [50].

Increased Tau phosphorylation is a common response to stress conditions that, if maintained over time and propagated in the brain can lead to the development of neurodegenerative diseases associated with cognition disorders known as tauopathies [28, 29]. In addition to the accumulation of ZIKV RNA in neurons and the induction of a DAM-like phenotype, we found that ZIKV infection induced a pathological phosphorylation of Tau in vitro and in vivo. The clusters of neurons displaying AT8 labeling in ZIKV-infected mice brain were surrounded by active microglial cells in a way reminiscent of what has been observed in AD, in post-mortem human hippocampus samples labeled with the AT8 antibody [51] and in brains of AD-like transgenic rat models [52]. It also resembled the way active microglial cells were shown to surround ZIKV-infected neurons in the brain of adult mice displaying permanent ZIKV replication [44]. A role for viral infection in AD etiology has been proposed [53] and Tau phosphorylation has been linked to viral infections such as herpes simplex virus (HSV) infection [54] and more recently to coronavirus disease 2019 (COVID-19) [55]. Many neurotropic virus infections have been identified as causing not only immediate but also delayed neuropathologies [56]. However, the biological mechanisms that underlie virus-induced delayed neuropathologies are for the most not understood. If maintained over time, ZIKV-induced pTau could be a factor participating in the development of delayed neuropathologies of the CNS such as those induced by ZIKV infection that are often observed after the resolution of infection [10–13, 16]. A role for active pro-inflammatory microglia in inducing pTau has been shown [57]. However, results obtained here in vitro with ZIKV-infected PCNs demonstrated that ZIKV infection could induce pTau independently of microglia.

In AD, the RNA-dependent protein kinase R (PKR) has been proposed as a candidate kinase responsible for the activation of glycogen synthase kinase B (GSK3B) [58, 59], which is one of the main kinases responsible for pTau [28, 29, 60, 61]. Even though delayed, the IFNB response induced by ZIKV in mature neurons triggered the expression of the gene coding for PKR that occurred concomitantly with the accumulation of viral RNA that induces PKR activation [62, 63]. Thus, ZIKV infection of neurons sets up the conditions for a PKR-dependent activation of GSK3B and the subsequent induction of pTau, potentially linking IFNB response to ZIKV-induced pTau. Preliminary results obtained in vitro (Additional file 5: Fig. S5) show that ZIKV infection indeed leads to a significant increase of the active form of kinase GSK3B in PCNs but not MEFs. However, further investigation is required to characterize the kinases involved in pathological Tau phosphorylation following ZIKV infection in mouse brain.

Overall, results presented here highlight the potential risks of a delayed virus-induced neuronal IFNB response that while being unable to totally stop viral replication can lead to the induction of deleterious effects in relation with the activation of the expression of neurotoxic factors by microglia and the induction of pTau in ZIKV-infected neurons. Further experiments are required to analyze the persistence of these early effects that have been observed here in the context of ZIKV infection but, more broadly, could also be implemented in response to many other viral infections that reach the CNS.

**Fig. 9:**
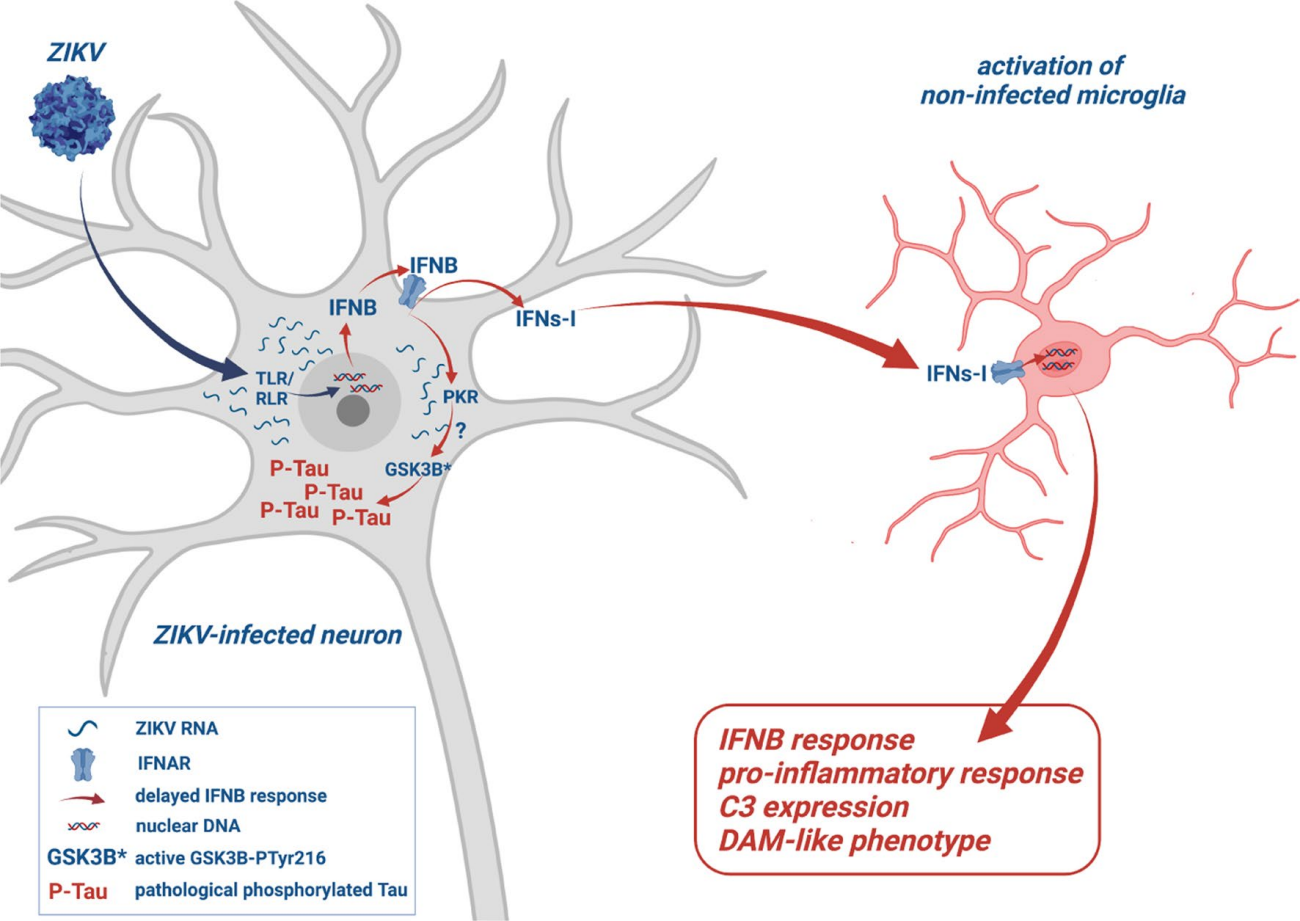
ZIKV-infection of neurons signals for IFNB-dependent microglia activation while inducing Tau phosphorylation. ZIKV-infected mature neurons from immunocompetent mice display a delayed IFNB response unable to stop viral replication leading to the accumulation of viral RNA in association with the induction of pathological phosphorylated Tau protein (P-Tau). ZIKV-infection leads to the activation of the expression of the gene coding for the RNA-dependent kinase PKR in PCNs and mouse brain. A role for PKR in the enhancement of P-Tau is proposed in relation with the capacity of PKR to be activated by viral cytoplasmic RNA and in turn activate GSK3B, one of the main kinases responsible of pTau. Following ZIKV infection of neurons, neuronal IFNs-I directly signal microglia for activation and the establishment of a DAM-like phenotype. The diagram was created using BioRender.com

Although different from human ZIKV infection, our cellular and animal models have provided insights into how mature neurons respond to ZIKV infection (summarized in Fig. 9) and highlight the value of the CC071 mouse strain as a new experimental model to investigate the cellular and molecular mechanisms underlying the development of virus-induced CNS pathologies associated with cognitive disorders.

## Conclusions

Our results obtained while analyzing the effect of ZIKV on mature neurons from immunocompetent mice in vitro, in PCNs and in vivo following IC inoculation of ZIKV show that neurons but not microglia constitute the cell type selectively infected by ZIKV and this even in the case of mice susceptible to ZIKV-induced disease. We show here that ZIKV-infected neurons display an inability to stop viral replication as a consequence of a delayed IFNB expression and response, which could be related with the capacity of ZIKV to target kinase TBK1 [64] that phosphorylates transcription factor IRF3 necessary to drive *Ifnb1* expression. IFNs-I secreted by ZIKV-infected neurons at late times p.i. then signal microglia for activation and secretion of pro-inflammatory, potentially neurotoxic factors. In vivo, uninfected microglial cells adopt an active morphology and a DAM-like expression profile, surrounding and sometimes engulfing neurons. In addition, ZIKV infection of neurons leads to the accumulation of pathological phosphorylated Tau protein in vitro and in vivo, reflecting a tauopathy-like phenotype. Overall, our results highlight the neurotoxic effects, potentially harmful for the CNS, deriving from the ZIKV infection of immunocompetent adult neurons.

## Supplementary Information

**Additional file 1.** **Fig. S1a.** Primary cultured neurons (PCNs) respond to ZIKV infection differently from murine embryonic fibroblasts (MEFs) of the same origin. Production of viral particles continuously increased throughout infection in ZIKV-infected PCNs but not MEFs. PCNs and MEFs from B6 embryos were infected with ZIKV at MOI=5. The number of ZIKV particles accumulated in MEF and PCN culture media was titrated using the focus-forming unit (FFU) assay at different times p.i. Symbols represent values obtained from independent experiments. **Fig. S1b.** The infection of primary cultured neurons (PCNs) with the Newcastle Disease Virus (NDV) induces an early IFNB expression and response. PCNs from B6 embryos were either non-infected (NI) or infected with NDV. The relative expression level of genes coding for IFNB (Ifnb1) and ISGs (Irf7, Ifna4, Eif2ak2, Oas1b) was analyzed by RT-qPCR with respect to Rplp0 used as reference gene. Symbols represent values obtained from independent infections of three independent primary cultures.

**Additional file 2.** Fig. S2. ZIKV-infection induces the activation of microglia in the brain of immunocompetent CC071 mice. CC001 and CC071 mice (5-6 week old) were necropsied at day 6 following IC inoculation of either PBS or 105 FFU of ZIKV. (**a**) As compared to non-susceptible CC001 mice, ZIKV infection of susceptible CC071 mice induced a significantly higher expression of ZIKV RNAs as determined by RT-qPCR analysis of total brain extracts with respect to Hrpt1 used as reference gene. Symbols represent individual mice. Data from n=5 CC001 and CC071 mice respectively are means without s.d. with significance assessed by two-way ANOVA Tukey’s multiple comparisons test. P-value <0.0001 (****) and ns= not significant. (**b**, **c**) ZIKV induced microglia activation in CC071 mice brains as determined by immunofluorescence and confocal microscopy with neurons labeled with anti-NeuN (green) and microglial cells with anti-Iba1 antibody (red). Single confocal sections (z projection) and the corresponding merge images are shown. Scale bars in (**b**)= 20 µm and (c)= 10 µm.

**Additional file 3.** **Fig. S3.** ZIKV infection does not induce pathological Tau phosphorylation recognized by AT8 antibodies in the brain of CC001 mice. CC001 and CC071 mice (5-6 week old) were necropsied at day 6 following IC inoculation of either PBS or 105 FFU of ZIKV. No induction of pTau recognized by AT8 antibodies was observed in the brain of CC001 mice following ZIKV infection as compared to PBS treated CC001 mice as determined by immunofluorescence and confocal microscopy with nuclear DNA labeled with DAPI (blue), pTau labeled with the AT8 antibody (green) and microglial cells labeled with anti-Iba1 antibody (red). Single confocal sections (z projection) and the corresponding merge images are shown. Scale bars= 20 µm.

**Additional file 4.** **Fig. S4.** ZIKV infection does not induce pathological Tau phosphorylation recognized by AT100 and AD2 antibodies in the brain of CC001 and CC071 mice. CC001 and CC071 mice (5-6 week old) were necropsied at day 6 following IC inoculation of either PBS or 105 FFU of ZIKV. No induction of pTau recognized by AT100 and AD2 antibodies was observed in the brain of ZIKV- as compared to PBS-treated mice as determined by immunofluorescence and confocal microscopy with nuclear DNA labeled with DAPI (blue), pTau labeled with (**a**) the AT100 or (**b**) AD2 antibodies (green). Single confocal sections (z projection) and the corresponding merge images are shown. Scale bars= 50 µm.

**Additional file 5.** **Fig. S5.** ZIKV infection induces the activation of the kinase GSK3B in PCNs but not MEFs. PCNs and MEFs from B6 embryos were either non-infected (NI) or ZIKV-infected (ZIKV) at a MOI=5. (**a**, **b**) ZIKV-infected PCNs displayed increased levels of the phosphorylated (phTyr216) active form of GSK3B as shown by (**a**) Western blot analysis, and (**b**) the corresponding quantification of total protein extracts collected from n (independent experiments) =4 for 48, =2 for 32 and =1 for 17 and 64 h p.i.. Data are means ± s.d. with significance assessed by one-way ANOVA Dunnett’s multiple comparison test with each condition compared to NI. P-value <0.01 (**), <0.05 (*) and ns= not significant.

**Additional file 6.** **Table S1.** Oligonucleotides.

## Data Availability

The data that support the finding of this study are available from the corresponding authors, upon reasonable request.

## Abbreviations

AD: Alzheimer’s disease
ANOVA: Analysis of variance
APOE: Apolipoprotein E
ARG1: Arginase 1
AXL: Axl receptor tyrosine kinase
B6: C57BL/6J
BDNF: Brain-derived neurotrophic factor
CC: Collaborative cross
CCL: Chemokine (C–C motif)
CLEC7a: C-type lectin domain containing 7a
CM: Conditioned medium
CNS: Central nervous system
CXCL: Chemokine (CXC motif) ligand
CXCR: Chemokine (CXC motif) receptor
DAM: Disease associated microglia
DIV: Days in vitro
d.p.i.: Days post-infection
FFA: Focus forming assay
FFU: Foci forming units
FBS: Fetal bovine serum
GSK3B: Glycogen synthase kinase 3B
IBA1: Ionized calcium-binding adapter molecule 1
IC: Intracranial
IFN: Interferon
IFNAR: Interferon alpha/beta receptor
Ifnb1: Murine interferon beta gene
IL: Interleukin
iNOS: Inducible nitric oxyde synthase
IP: Intraperitoneal
IRF: Interferon regulatory factor
ISG: Interferon inducible gene
MAP2: Microtubule associated protein 2
MEF: Murine embryonic fibroblasts
MEF2c: Myocyte-specific enhancer factor 2c
MOI: Multiplicity of infection
NeuN: Neuronal nuclear protein
NI: Non-infected
NS2B: Non-structural protein 2B
NT: Non-treated
OAS: Oligoadenylate synthethase
P2RY12: Purigenic receptor P2Y12
p.i.: Post-infection
PCN: Primary cultured neurons
PBS: Phosphate-buffered saline
PKR: Protein kinase R
PRR: Pattern recognition receptors
pTau: Pathological phosphorylation of Tau protein
recIFNB: Recombinant interferon beta
RIG: Retinoic acid-inducible gene
SALL1: Spalt like transcription factor 1
s.d.: Standard deviation
STAT: Signal transducer and activator of transcription
TGFb: Transforming growth factor beta
TNFa: Tumor necrosis factor a
TREM2: Triggering receptor expressed on myeloid cells 2
ZIKV: Zika virus
