# Supplementary figures and images for: Zika virus infection of mature neurons from immunocompetent mice generates a disease-associated microglia and a tauopathy-like phenotype in link with a delayed interferon beta response
Source: J Neuroinflammation. 2022 Dec 20;19:307. doi: 10.1186/s12974-022-02668-8 (PMC9764315; doi:10.1186/s12974-022-02668-8)

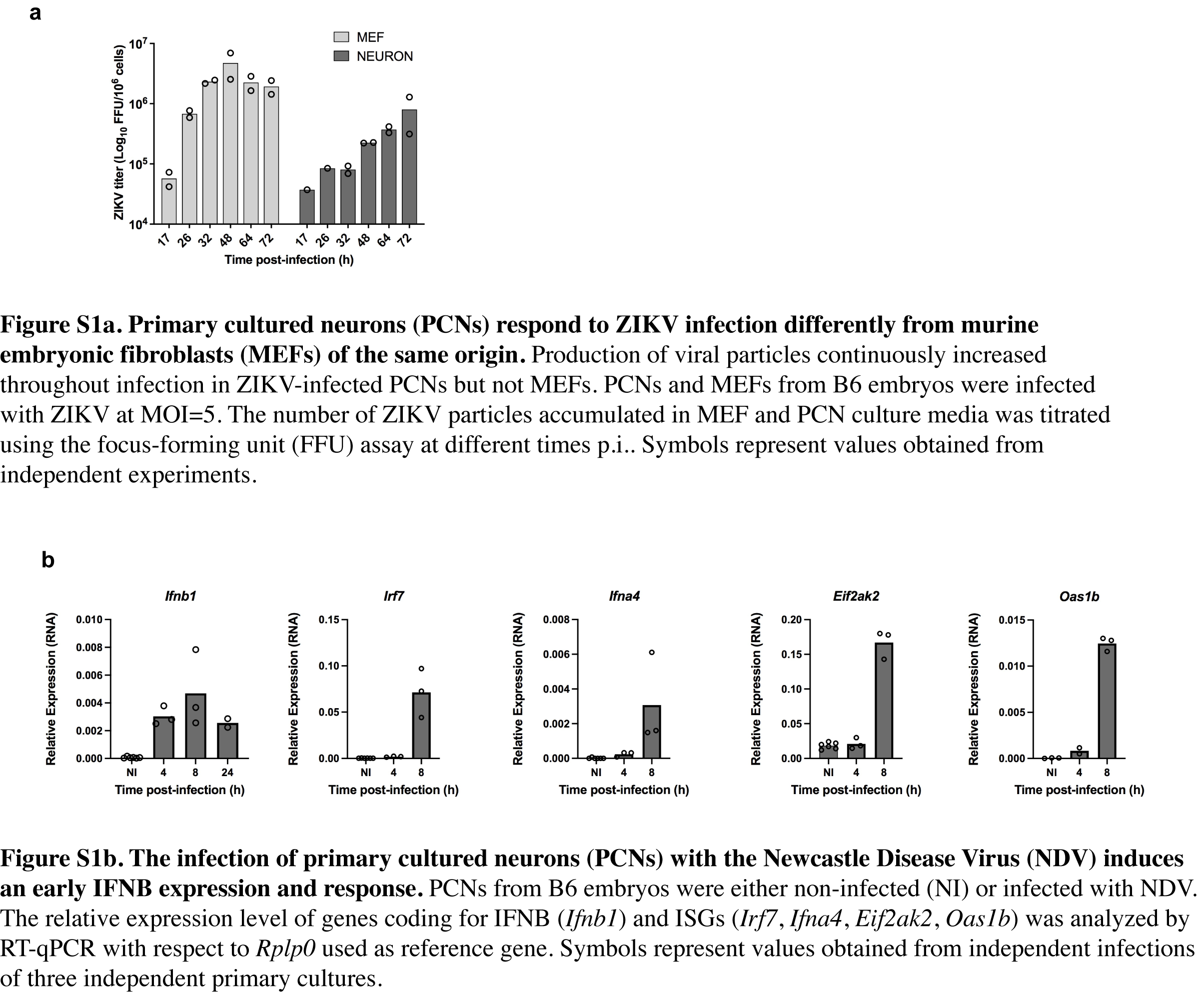

Supplement: Supplementary file 1 — Additional file 1. Fig. S1a. Primary cultured neurons (PCNs) respond to ZIKV infection differently from murine embryonic fibroblasts (MEFs) of the same origin. Production of viral particles continuously increased throughout infection in ZIKV-infected PCNs but not MEFs. PCNs and MEFs from B6 embryos were infected with ZIKV at MOI=5. The number of ZIKV particles accumulated in MEF and PCN culture media was titrated using the focus-forming unit (FFU) assay at different times p.i. Symbols represent values obtained from independent experiments. Fig. S1b. The infection of primary cultured neurons (PCNs) with the Newcastle Disease Virus (NDV) induces an early IFNB expression and response. PCNs from B6 embryos were either non-infected (NI) or infected with NDV. The relative expression level of genes coding for IFNB (Ifnb1) and ISGs (Irf7, Ifna4, Eif2ak2, Oas1b) was analyzed by RT-qPCR with respect to Rplp0 used as reference gene. Symbols represent values obtained from independent infections of three independent primary cultures. [file 12974_2022_2668_MOESM1_ESM.tif]

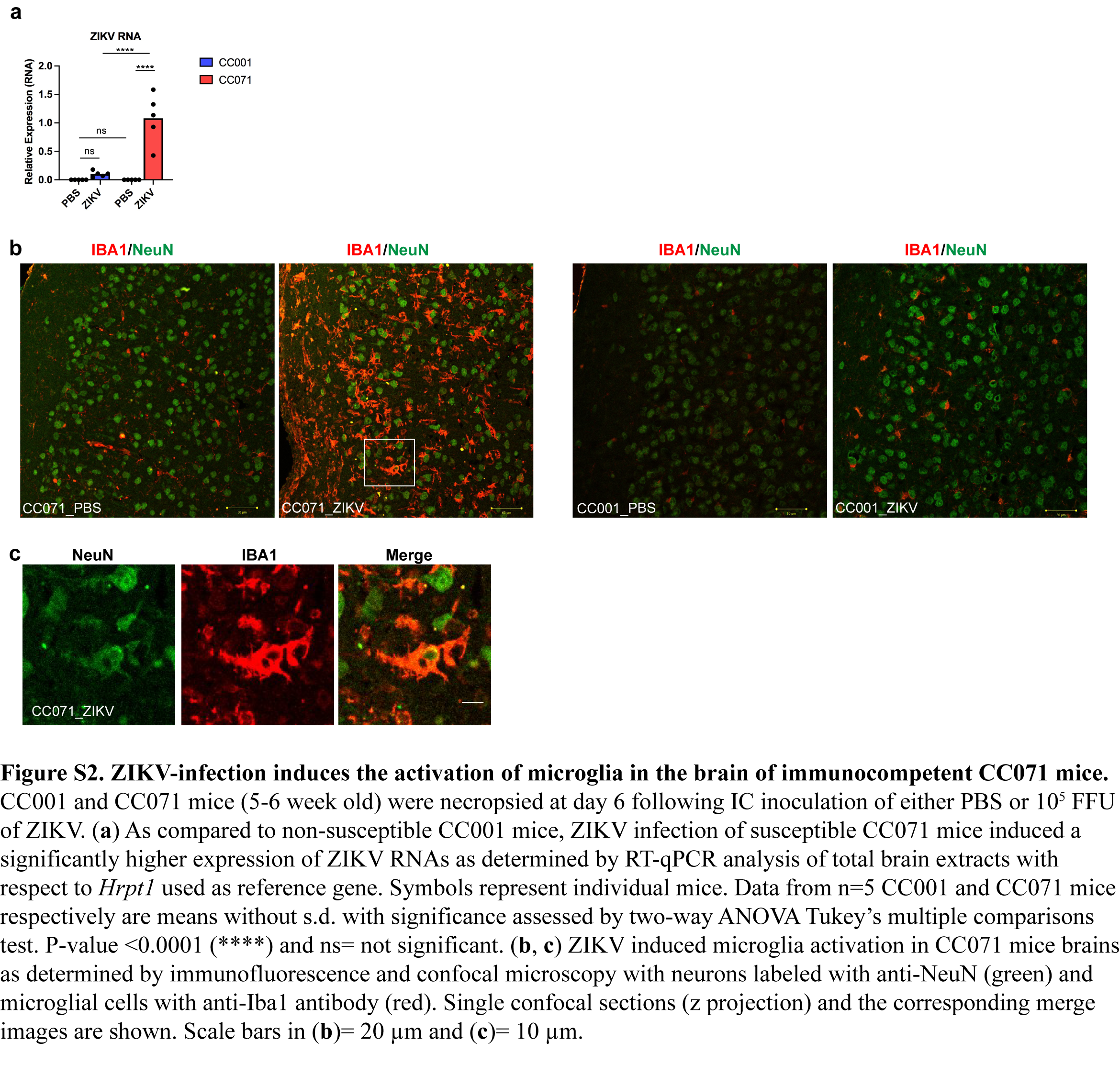

Supplement: Supplementary file 2 — Additional file 2. Fig. S2. ZIKV-infection induces the activation of microglia in the brain of immunocompetent CC071 mice. CC001 and CC071 mice (5-6 week old) were necropsied at day 6 following IC inoculation of either PBS or 105 FFU of ZIKV. (a) As compared to non-susceptible CC001 mice, ZIKV infection of susceptible CC071 mice induced a significantly higher expression of ZIKV RNAs as determined by RT-qPCR analysis of total brain extracts with respect to Hrpt1 used as reference gene. Symbols represent individual mice. Data from n=5 CC001 and CC071 mice respectively are means without s.d. with significance assessed by two-way ANOVA Tukey’s multiple comparisons test. P-value <0.0001 (****) and ns= not significant. (b, c) ZIKV induced microglia activation in CC071 mice brains as determined by immunofluorescence and confocal microscopy with neurons labeled with anti-NeuN (green) and microglial cells with anti-Iba1 antibody (red). Single confocal sections (z projection) and the corresponding merge images are shown. Scale bars in (b)= 20 µm and (c)= 10 µm. [file 12974_2022_2668_MOESM2_ESM.tif]

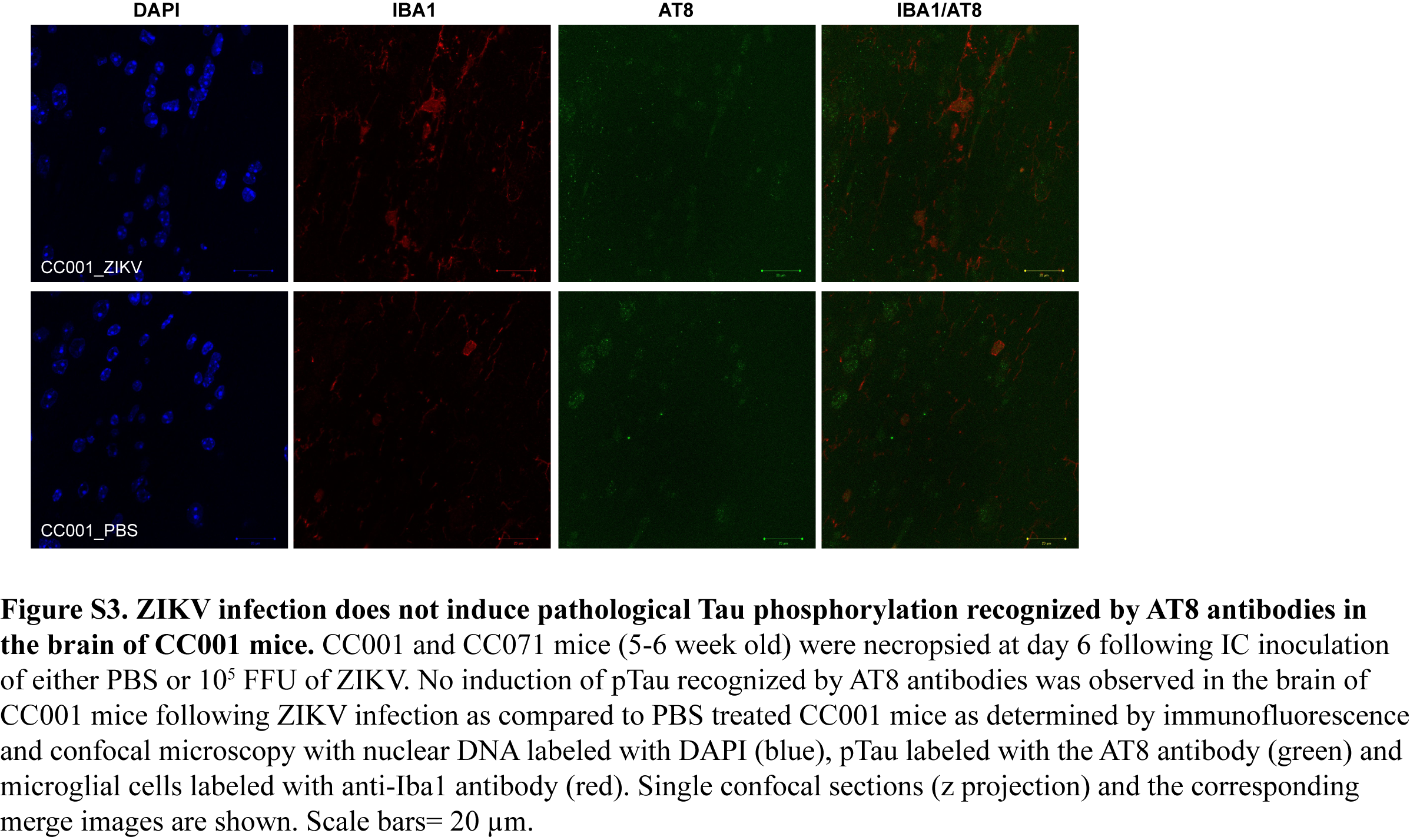

Supplement: Supplementary file 3 — Additional file 3. Fig. S3. ZIKV infection does not induce pathological Tau phosphorylation recognized by AT8 antibodies in the brain of CC001 mice. CC001 and CC071 mice (5-6 week old) were necropsied at day 6 following IC inoculation of either PBS or 105 FFU of ZIKV. No induction of pTau recognized by AT8 antibodies was observed in the brain of CC001 mice following ZIKV infection as compared to PBS treated CC001 mice as determined by immunofluorescence and confocal microscopy with nuclear DNA labeled with DAPI (blue), pTau labeled with the AT8 antibody (green) and microglial cells labeled with anti-Iba1 antibody (red). Single confocal sections (z projection) and the corresponding merge images are shown. Scale bars= 20 µm. [file 12974_2022_2668_MOESM3_ESM.tif]

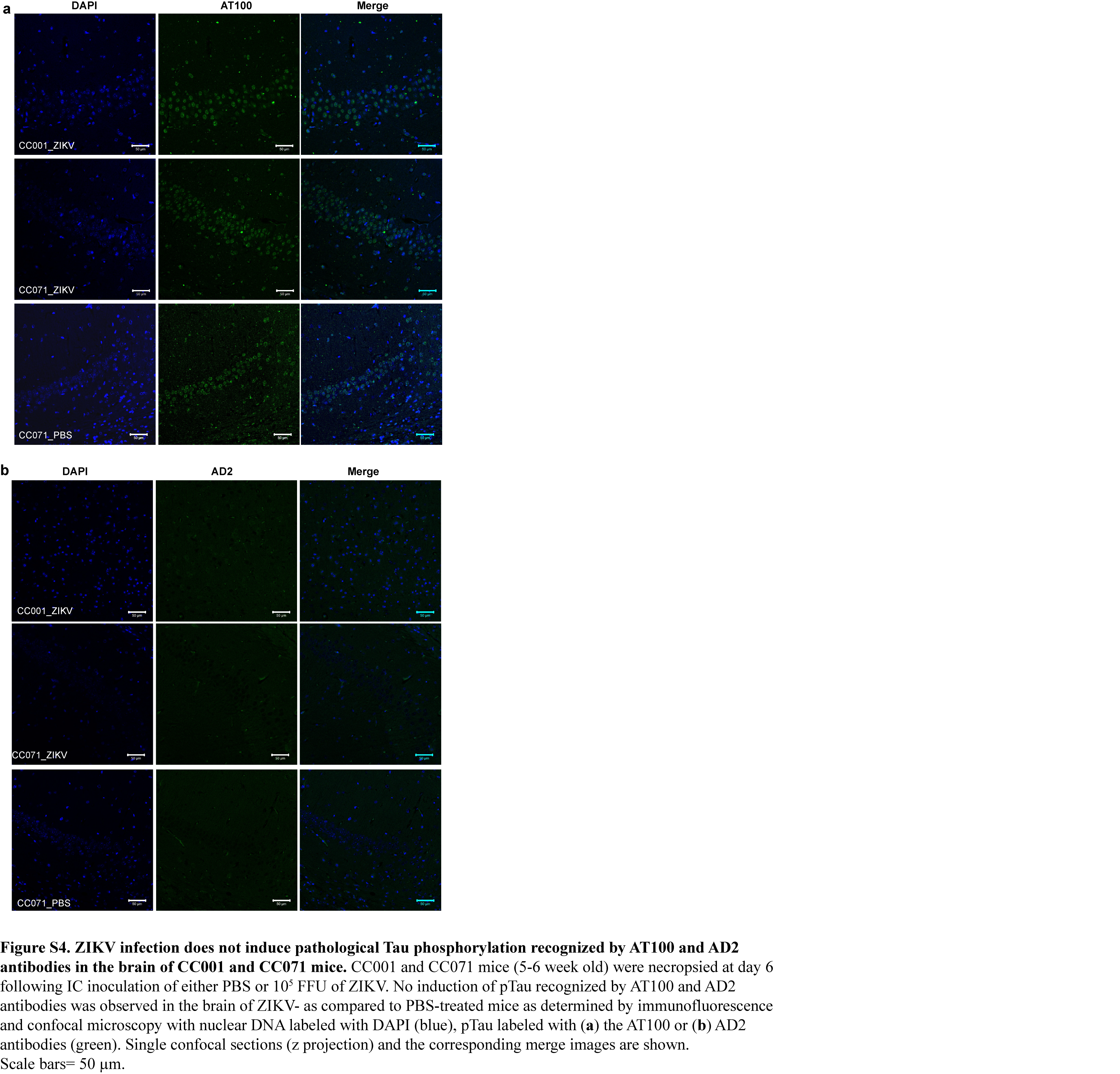

Supplement: Supplementary file 4 — Additional file 4. Fig. S4. ZIKV infection does not induce pathological Tau phosphorylation recognized by AT100 and AD2 antibodies in the brain of CC001 and CC071 mice. CC001 and CC071 mice (5-6 week old) were necropsied at day 6 following IC inoculation of either PBS or 105 FFU of ZIKV. No induction of pTau recognized by AT100 and AD2 antibodies was observed in the brain of ZIKV- as compared to PBS-treated mice as determined by immunofluorescence and confocal microscopy with nuclear DNA labeled with DAPI (blue), pTau labeled with (a) the AT100 or (b) AD2 antibodies (green). Single confocal sections (z projection) and the corresponding merge images are shown. Scale bars= 50 µm. [file 12974_2022_2668_MOESM4_ESM.tif]

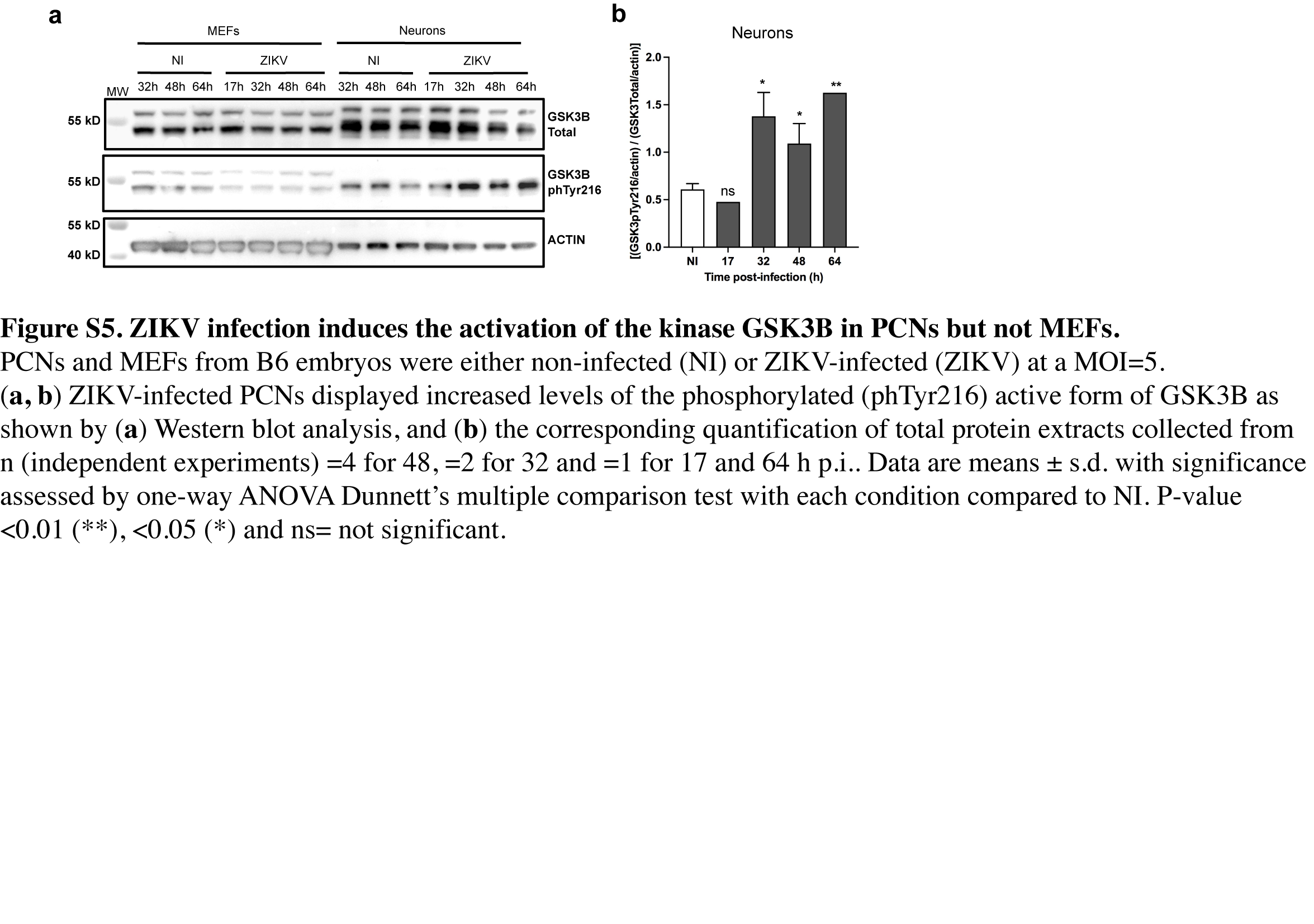

Supplement: Supplementary file 5 — Additional file 5. Fig. S5. ZIKV infection induces the activation of the kinase GSK3B in PCNs but not MEFs. PCNs and MEFs from B6 embryos were either non-infected (NI) or ZIKV-infected (ZIKV) at a MOI=5. (a, b) ZIKV-infected PCNs displayed increased levels of the phosphorylated (phTyr216) active form of GSK3B as shown by (a) Western blot analysis, and (b) the corresponding quantification of total protein extracts collected from n (independent experiments) =4 for 48, =2 for 32 and =1 for 17 and 64 h p.i.. Data are means ± s.d. with significance assessed by one-way ANOVA Dunnett’s multiple comparison test with each condition compared to NI. P-value <0.01 (**), <0.05 (*) and ns= not significant. [file 12974_2022_2668_MOESM5_ESM.tif]

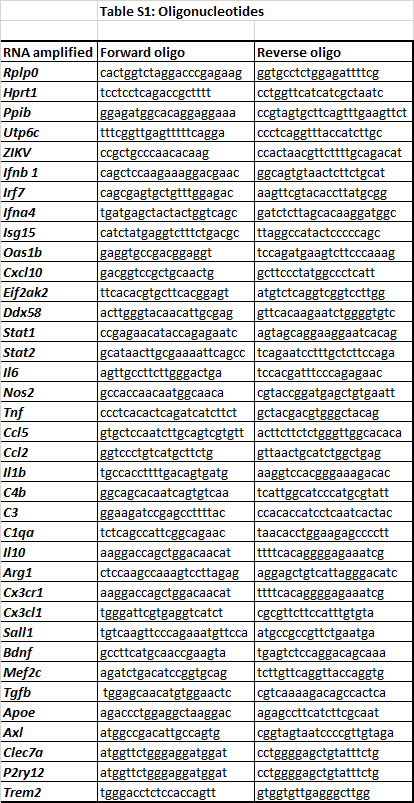

Supplement: Supplementary file 6 — Additional file 6. Table S1. Oligonucleotides. [file 12974_2022_2668_MOESM6_ESM.tif]
